# Cooperative inhibition in cytochrome P450 between a substrate and an apparent noncompetitive inhibitor

**DOI:** 10.1016/j.jbc.2025.108513

**Published:** 2025-04-15

**Authors:** Yu Hirano, Sachiyo Yoneda, Kaori Yasuda, Noriyuki Kurita, Fumihiro Kawagoe, Bunzo Mikami, Teisuke Takita, Kiyoshi Yasukawa, Shinichi Ikushiro, Midori Takimoto-Kamimura, Atsushi Kittaka, Toshiyuki Sakaki, Taro Tamada

**Affiliations:** 1 Institute for Quantum Life Science, National Institutes for Quantum Science and Technology, Chiba, Japan; 2 Quantum Life Science Course, Graduate School of Science and Engineering, Chiba University, Chiba, Japan; 3 Graduate School of Engineering, Biotechnology and Pharmaceutical Engineering, Toyama Prefectural University, Toyama, Japan; 4 Department of Computer Science and Engineering, Toyohashi University of Technology, Toyohashi, Aichi, Japan; 5 Faculty of Pharmaceutical Sciences, Teikyo University, Itabashi, Tokyo, Japan; 6 Research Institute for Sustainable Humanosphere, Kyoto University, Kyoto, Japan; 7 Institute of Advanced Energy, Kyoto University, Kyoto, Japan; 8 Division of Food Science and Biotechnology, Graduate School of Agriculture, Kyoto University, Kyoto, Japan; 9 Quantum-Structural Life Science Laboratories, CBI Research Institute, Tokyo, Japan

**Keywords:** crystal structure, cytochrome P450, enzyme kinetics, heme, inhibition mechanism, ESI complex, quantum mechanical calculation

## Abstract

Cytochrome P450 (CYP) enzymes bind a heme group that acts as a catalytic center. Inhibition mechanisms in CYP enzymes have been studied extensively by biochemical and structural analyses. Noncompetitive inhibitors are generally believed to bind to allosteric sites remote from the active site to form enzyme–substrate–inhibitor (ESI) complexes. Docking simulations predict the binding sites of noncompetitive inhibitors to CYP enzymes, but to date, there has been no experimental structural verification of ESI complexes formed by CYP enzymes. We performed biochemical and structural analyses of CYP105A1 using the imidazole-containing inhibitors ketoconazole, lanoconazole, and miconazole. Enzyme inhibition analyses demonstrated that ketoconazole and miconazole act as competitive inhibitors, whereas lanoconazole acts as a noncompetitive inhibitor of CYP105A1. The obtained X-ray structures of enzyme-inhibitor (EI) complexes showed that lanoconazole can bind in various orientations to the heme iron compared with ketoconazole and miconazole. We also determined the X-ray structure of an ESI complex comprising CYP105A1, diclofenac, and lanoconazole. This structure shows that lanoconazole binds to the heme iron and that diclofenac closely interacts with the bound lanoconazole, but it is positioned distant from the heme group. Quantum mechanical calculations indicate that Cl-π and electrostatic interactions between diclofenac and lanoconazole, and electrostatic interactions between diclofenac and positively charged arginine residues, stabilize the formation of the ESI complex. Based on these results, we propose a mechanism for cooperative inhibition between a substrate and an apparent noncompetitive inhibitor.

Cytochrome P450 (CYP) is a large family of heme-containing enzymes. The heme iron atom functions as a catalytic center for various reactions, such as the metabolism of xenobiotics, the metabolism of lipids, and the synthesis of hormones. The heme group resides in a substrate-binding pocket. The properties of the pocket regulate the selection of the substrate and the reaction at the active sites. CYP inhibitors have important roles for clinical applications; for example, CYP3A4 inhibitors include many drugs and some of them cause drug-drug interactions (1), CYP19A1 inhibitors can be used to treat estrogen-dependent breast cancer (2), and CYP51 inhibitors are used as antifungal agents (3). The biochemical mechanisms of CYP inhibition have been studied intensively experimentally (4), whereas our understanding of the molecular mechanisms of inhibition effects relies on the three-dimensional structural information gleaned from over nine hundred CYP protein structures in the Protein Data Bank (PDB).

Novel CYP enzyme inhibitors are generally designed based on the structures of known inhibitors bound in the active site (5, 6, 7). Inhibitors containing an azole group bind to the heme iron (2, 5, 8) and compete with the substrate to bind to the active site (4, 9). Such competitive inhibition indicates that the substrate or the inhibitor can bind to the active site. There are also CYP noncompetitive inhibitors (10, 11, 12, 13) that likely bind to allosteric sites that are distant from the active site of the enzyme (4, 9). This mechanism of inhibition likely inhibits structural changes in the enzyme required for the reaction to proceed. Furthermore, a noncompetitive inhibitor has the same affinity for the enzyme (E) and for the enzyme-substrate (ES) complex and that substrate has the same affinity for E and for the enzyme-inhibitor (EI) complex. Noncompetitive inhibitors are thus believed to form an enzyme-substrate-inhibitor (ESI) complex. A previous docking simulation predicted the binding site of noncompetitive inhibitors in CYP3A4 (13). The docking models showed that the inhibitors bound to an allosteric site formed by the surface loops of CYP3A4. The docking simulation assumed that noncompetitive inhibitors bind to this allosteric site and not to the active site. Inhibition of CYP2D6 by quinidine has been well described by the two-site inhibition model in which a substrate and an inhibitor are thought to form an ESI complex (14). The X-ray crystal structure of CYP2D6 and quinidine complex has shown that quinidine is bound near the entrance channel in the binding pocket and distal from the heme group (15). However, to date, there has been no experimental structural evidence of an ESI complex formed by CYP enzymes, and thus the molecular mechanisms underlying noncompetitive inhibition remain unclear, despite extensive biochemical and structural analyses of inhibition effects on CYP enzymes.

In this work, we selected CYP105A1 from *Streptomyces griseolus* to study the biochemical and structural mechanisms of inhibition (16, 17). CYP105A1 can catalyze 2-step (25- and 1α-) hydroxylation reactions of vitamin D_3_ (VD_3_) to produce hormonally active 1α,25-dihydroxyvitamin D_3_ (1α,25(OH)_2_D_3_). The X-ray crystal structures of CYP105A1 have been determined at high resolution (1.5–2.0 Å) for the substrate-free enzyme and for the 1α,25(OH)_2_D_3_ complex (18, 19). The structures show that the ligand binding pocket is predominantly surrounded by hydrophobic residues, although there are three arginine residues (Arg73, Arg84, and Arg193) in the binding pocket. The mutation of Arg73 and Arg84 enhanced hydroxylation to provide VD_3_ derivatives. The Arg84Ala (R84A) mutant exhibited higher hydroxylation activity than the wild-type enzyme, with a *k*_cat_/*K*_m_ value > 30-fold higher for 25-hydroxylation and >10-fold higher for 1α-hydroxylation (19). Our previous studies showed that wild-type and mutant CYP105A1 metabolize 12 types of non-steroidal anti-inflammatory drugs (NSAIDs), including diclofenac (20), with the R84A mutant exhibiting particularly high activity towards many NSAIDs. We recently also found that the R84A variant shows high activity towards statins, which are mainly metabolized by human CYP3A4 (manuscript in preparation). The volume of the substrate pocket of CYP105A1 is much larger than that of microbial CYPs such as CYP101 and CYP102, and is similar to that of CYP3A4 (21), a representative human drug-metabolizing P450 enzyme. The high-resolution structural information available for CYP105A1 aids in understanding the molecular mechanisms of inhibition effects. We performed spectroscopic analyses to investigate the inhibition effects of azole-based inhibitors toward CYP105A1 and X-ray structural analyses of the EI and ESI complexes to understand the molecular mechanisms of the inhibition effects. Quantum mechanical molecular orbital calculations were conducted to elucidate the underlying features of the interactions in the ESI complex.

## Results

### Enzyme inhibition assay

**Figure 1:**
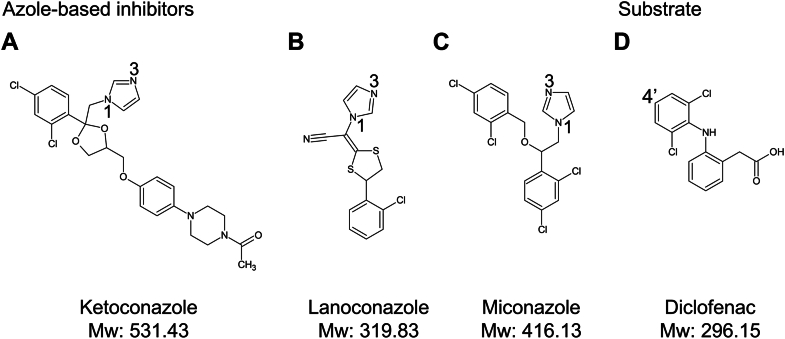
**Schematic representation of the chemical structures of the azole-based inhibitors and substrate used in this study.***A*, The inhibitors ketoconazole *B*, lanoconazole, and *C*, miconazole contain an imidazole group. Positions 1 and 3 of the nitrogens in the imidazole group are indicated *D*. Diclofenac is the substrate, and the 4′-hydroxylation site is indicated.

The azole-based inhibitors used for this study were commercially available antifungal drugs (ketoconazole, lanoconazole, miconazole) (22, 23, 24), and diclofenac was used as the substrate (Fig. 1). The UV-visible absorption spectrum of purified CYP105A1 R84A showed that the enzyme is in the ferric form, with a Soret band at 419 nm and α, β bands at 570 and 538 nm (Fig. S1*A*). Adding an inhibitor to the ferric form of CYP105A1 R84A caused type II red shifts of the Soret band to 421 to 424 nm (Fig. S1*B*), indicating that the heme iron was directly coordinated by the imidazole group of the inhibitor (25).

Table 1 shows the *K*_d_ (dissociation constant) values of CYP105A1 R84A for each inhibitor, calculated from the enzyme-inhibitor binding difference UV spectrum (Fig. S2*A* and S2*B*). The *K*_d_ values for ketoconazole, lanoconazole, and miconazole were 4.58 ± 0.76, 0.13 ± 0.10, and 0.32 ± 0.30 μM, respectively, indicating that lanoconazole has the highest affinity for CYP105A1 R84A, followed by miconazole, with ketoconazole having the lowest affinity.

**Table 1:** Kinetic parameters for each inhibitor on the hydroxylation of diclofenac by CYP105A1 R84A

Inhibitor	*K*_d_ (μM)	*K*_i_ (μM)
Ketoconazole	4.58 ± 0.76	22.68 ± 2.33
Lanoconazole	0.13 ± 0.10	0.90 ± 0.10
Miconazole	0.32 ± 0.30	1.20 ± 0.35

Each value is the mean ± standard deviation from three separate experiments.

**Figure 2:**
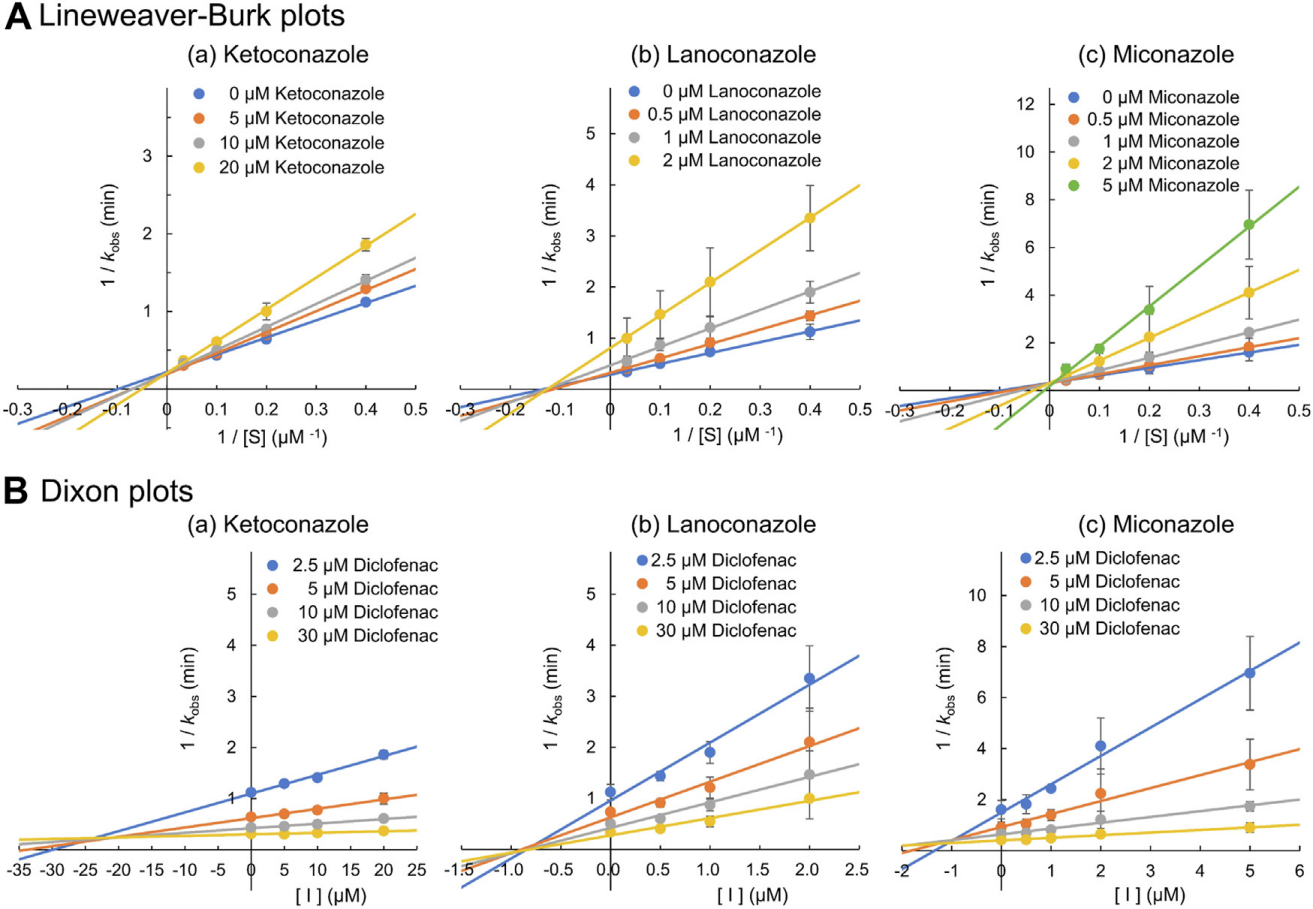
**Lineweaver-Burk plots (*A*) and Dixon plots (*B*) for each inhibitor.** The observed rate constant, kobs (min^−^^1^), is calculated as v/[E], where v is the reaction rate (μM/min), and [E] is the concentration (μM) of CYP105A1 R84A. The value for 30 μM diclofenac in 5 μM miconazole is the mean from two separate experiments. All other values are the mean from three separate experiments.

The inhibitory effect of each inhibitor on the diclofenac 4′-hydroxylation activity of CYP105A1 R84A was compared. Figure 2 shows the Lineweaver-Burk plot and Dixon plot for each inhibitor. Ketoconazole and miconazole clearly exhibited competitive inhibition, whereas lanoconazole exhibited noncompetitive inhibition. The *K*_i_ (inhibition constant) values for ketoconazole, lanoconazole, and miconazole calculated from their Dixon plots were 22.68 ± 2.33, 0.90 ± 0.10, and 1.20 ± 0.35 μM. These affinities for CYP105A1 R84A were consistent with their *K*_d_ values, although the *K*_i_ values were higher than the *K*_d_ values.

### Heme iron coordination in the EI complexes

The X-ray crystal structures were determined at 1.8 Å resolution for all CYP105A1 R84A-inhibitor complexes (Table S1). The m*F*_o_-D*F*_c_ polder omit map of each inhibitor indicated binding of the imidazole group to the heme iron (Fig. S3). The Cys355 thiolate is the common axial ligand of the heme iron in all the EI complexes studied here, with the nitrogen (position 3) of the imidazole group of each inhibitor coordinating at the sixth coordination position of the heme iron. The distance between the heme iron and the nitrogen of the imidazole group is 2.09 Å for ketoconazole, 2.10 Å for lanoconazole, and 2.11 Å for miconazole (Fig. S3). Ketoconazole was bound in a single conformation (Fig. S3*A*), whereas lanoconazole was bound in two conformations through highly different binding modes (Fig. S3*B*). The electron density of the chlorophenyl groups is slightly obscure, and thus their atomic positions could not be determined accurately, indicating that the chlorophenyl group has rotational flexibility in the binding pocket around the bond between the dithiolane and the chlorophenyl groups. Miconazole was also bound in two conformations, with the two dichlorophenyl groups in different orientations (Fig. S3*C*).

The overall protein structures of the EI complexes were highly similar (Fig. S4*A*). The root-mean-square-deviations (r.m.s.d.) for 400 C^α^ atoms were 0.30 to 0.35 Å (Table S2). The structure of the heme group was not significantly changed following binding of the inhibitor (Fig. S4*B*). The imidazole planes show different orientations for each inhibitor. Other parts of the inhibitors displayed different binding modes, including the positions of the chlorophenyl groups.

### Inhibitor binding pocket

**Figure 3:**
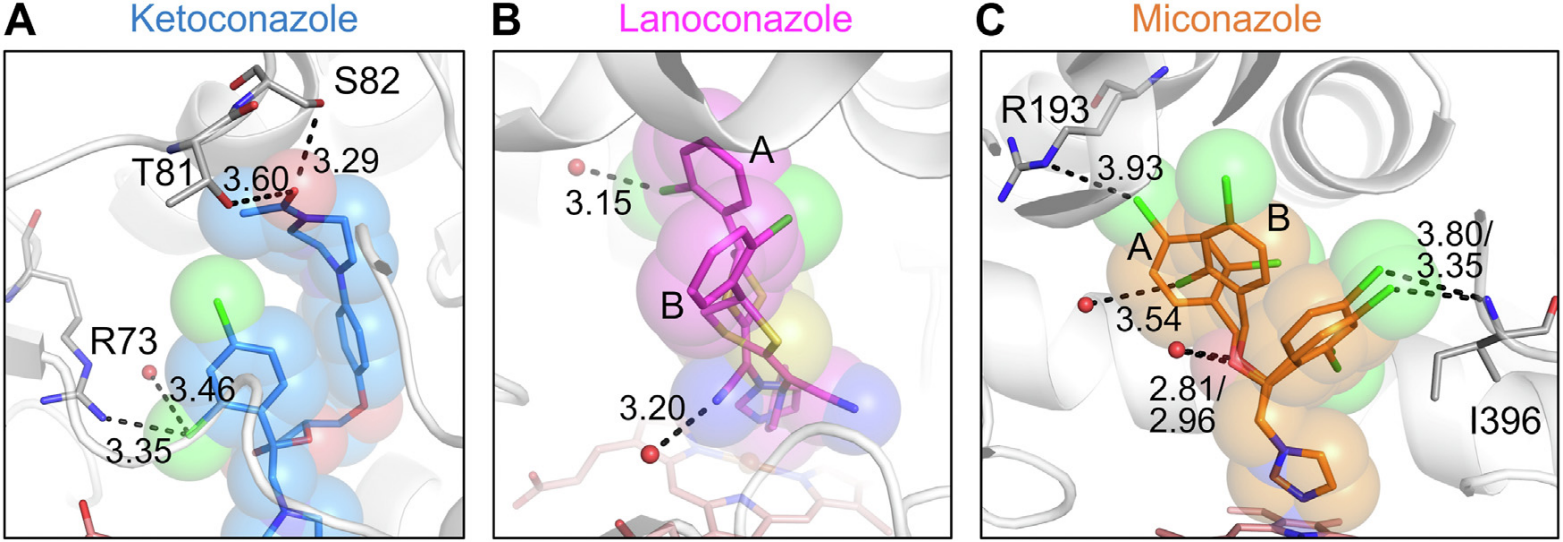
**Hydrophilic interactions in the inhibitor binding pocket of the EI complexes**. *A*, the ketoconazole binding site, *B*, the lanoconazole binding site, and *C*, the miconazole binding site. The inhibitors are shown as sticks, and the van der Waals radii of the inhibitor atoms are transparently overlayed. The residues and water molecules involved in the hydrophilic interactions are indicated in the figures. Hydrophilic interactions are shown as dotted lines with the distances in Å.

The bound inhibitors were generally surrounded by hydrophobic residues, with several polar (Thr81, Ser82, Ser181, Thr248, Thr394, and Thr395) and positively charged (Arg73 and Arg193) residues located within 4 Å of the bound inhibitor (Figs. S5 and S6*A*). Hydrophilic interactions between the inhibitor and the binding pocket are indicated by the bound inhibitor environment. In the ketoconazole complex, one chloride of the dichlorophenyl group is positioned 3.35 Å from the side chain N^η2^ of Arg73 and 3.46 Å from a water molecule, and the oxygen of the terminal acetyl group is 3.60 Å and 3.29 Å from the side-chain hydroxyl oxygens of Thr81 and Ser82, respectively (Fig. 3*A*). In the lanoconazole complex, the nitrogen of the nitrile group and the chloride of the chlorophenyl group in conformation A are 3.20 Å and 3.15 Å from two water molecules (Fig. 3*B*). In the miconazole complex, one chloride of the dichlorophenyl group of conformation A is 3.93 Å from the side chain N^ε^ of Arg193, one chloride in the dichlorophenyl group of conformation B is 3.54 Å from a water molecule, one chloride of the other dichlorophenyl group is 3.80/3.35 Å (conformation A/B) from the main chain amide nitrogen of Ile396, and the oxygen of the oxy group is 2.81/2.96 Å (conformation A/B) from a water molecule (Fig. 3*C*).

### Structural comparison of the binding pocket in the EI complexes

**Figure 4:**
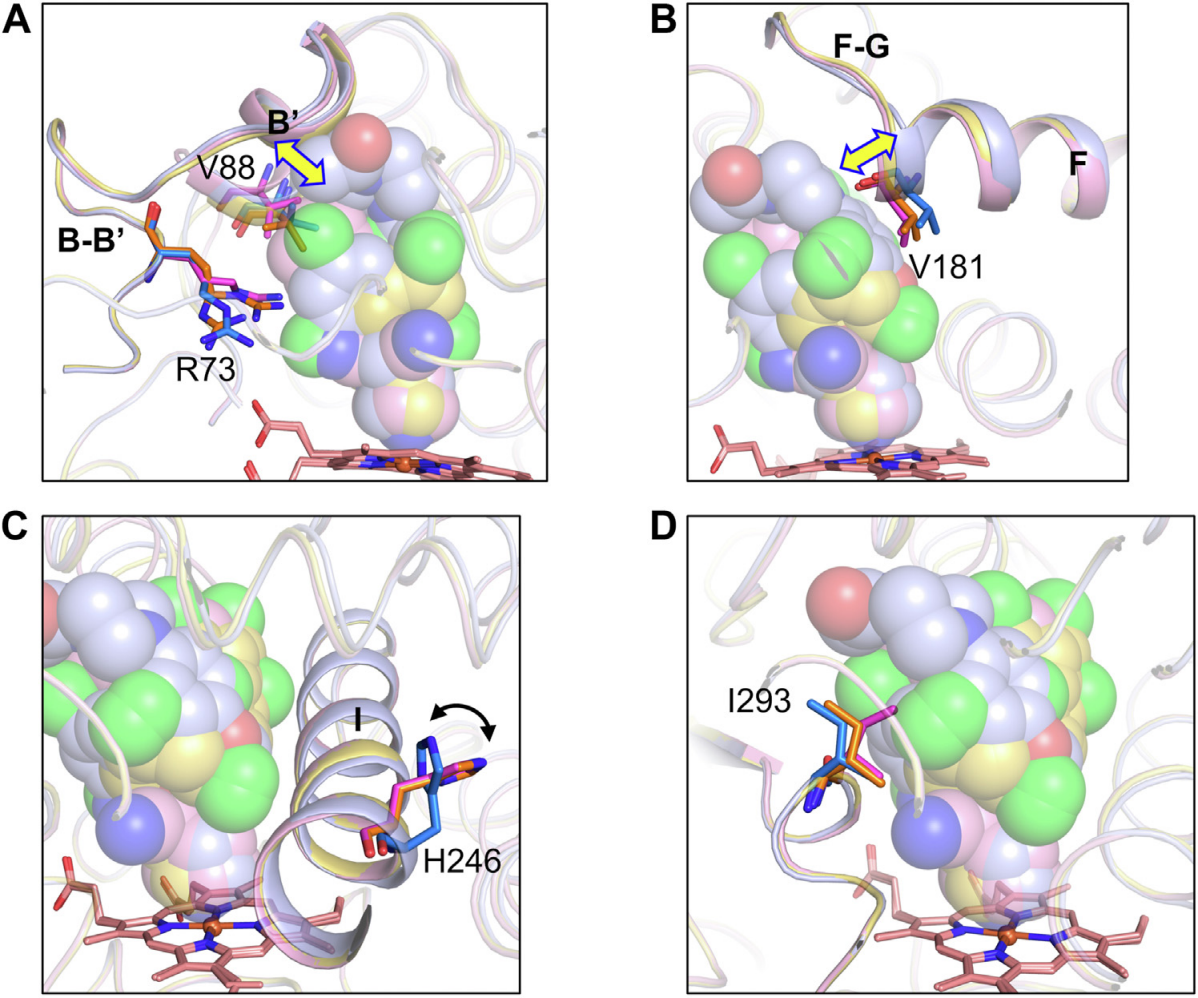
**Structural comparison of the binding pocket amongst the EI complexes**. *A*, the B′ helix and B-B′ loop, *B*, the F helix and F-G loop, *C*, the I helix, and *D*, the loop (Ala289-Gly295). The ketoconazole, lanoconazole, and miconazole complexes are colored *blue*, *pink*, and *orange*, respectively. Inhibitors are shown as CPK models, and amino acid residues indicated in the figures are shown as sticks. Structural differences in the B′ and F helices are indicated by *yellow* double arrows in (*A*) and (*B*). The distances of the Val88 C^α^ atom are 1.69 Å (ketoconazole-lanoconazole) and 0.60 Å (ketoconazole-miconazole), and those of the Val181 C^α^ atom are 0.78 Å (ketoconazole-lanoconazole) and 1.10 Å (ketoconazole-miconazole). The flip of the His246 side chain is indicated by a *black* double arrow in (*C*).

Although the positions of the B′ helices show variations in the EI complexes (Figs. 4*A* and S7*A*), Val88 in the B′ helix is located within 4 Å of each inhibitor (Fig. S6*A*). The positions of the main chain of Arg73 in the B-B′ loop are similar among the EI complexes, but the side chain of Arg73 adopts various conformations in the different complexes (Fig. 4*A*). The terminal residues of the F helix and the residues in the F-G loop are positioned differently in the ketoconazole complex compared with the lanoconazole and miconazole complexes (Figs. 4*B* and S7*B*) due to the phenyl group of ketoconazole being located near Val181 in the F helix. The positions of the G helix and of Arg193 in the helix are similar among the EI complexes (Fig. S8*A*). Four residues (Leu240, Ile243, Ala244, and Thr248) in the I-helix are within 4 Å of each inhibitor (Fig. S6*A*). The position of the I helix is similar amongst the EI complexes, but His246 is flipped in the ketoconazole complex (Figs. 4*C* and S7*C*). The E helix and Phe173 in the F helix located near His246 show positional differences in the ketoconazole complex compared with the lanoconazole and miconazole complexes (Fig. S8*B*). The loop containing Ile293 is within 4 Å of each inhibitor and the position of this loop is similar in each EI complex (Figs. 4*D* and S7*D*).

### The X-ray structure of the ESI complex

**Figure 5:**
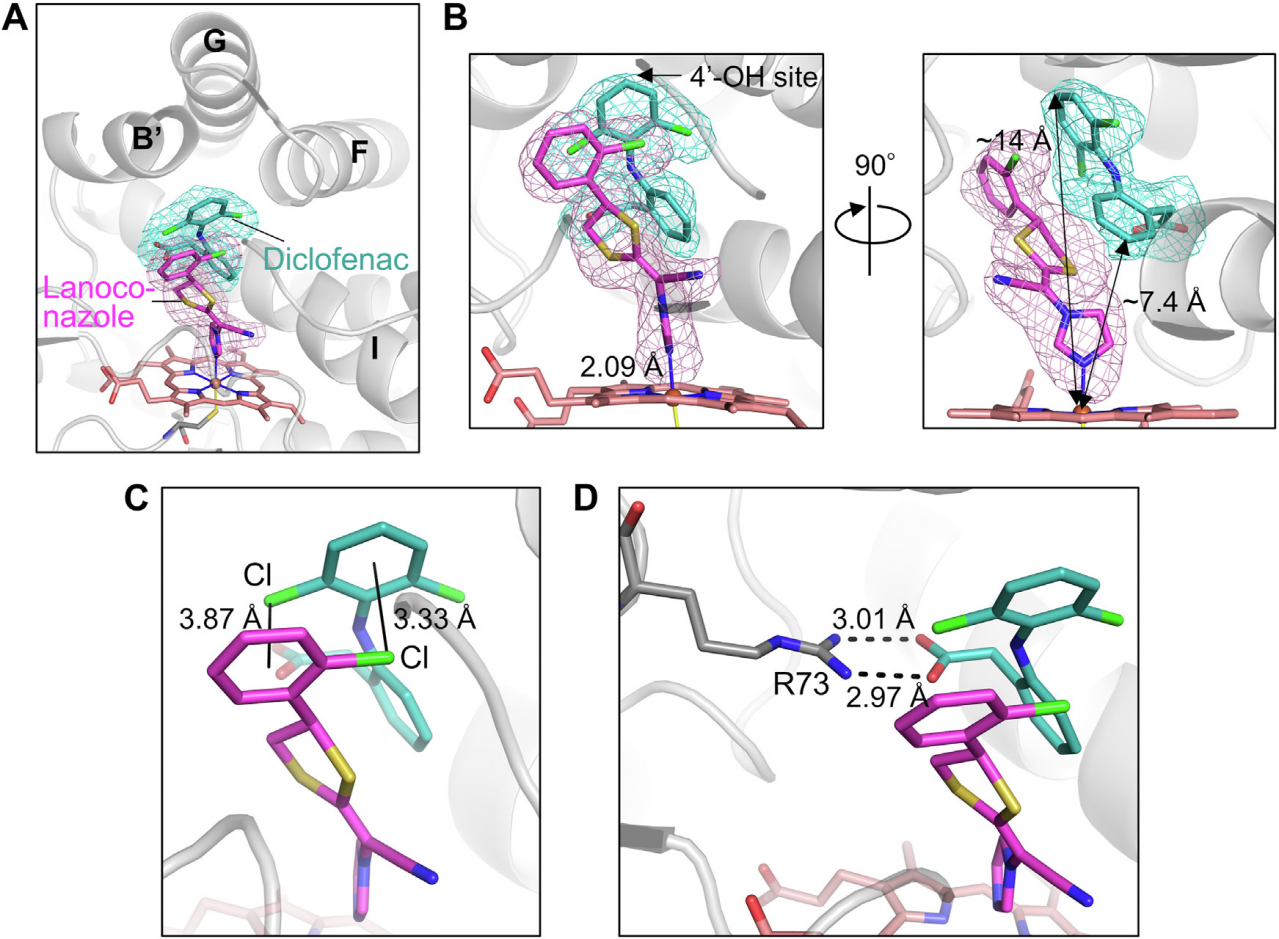
**X-ray crystal structure of the ESI complex**. *A*, mFo-DFc polder omit map of diclofenac and lanoconazole. The omit maps contoured at 3.0 σ levels are shown as a *light blue* mesh (for diclofenac) and *pink* mesh (for lanoconazole). Diclofenac and lanoconazole are shown as *blue-green* and *magenta* sticks, respectively. The Fe coordinate bonds are shown as thin lines. *B*, Close-up views of the binding site. The *right* panel is the view 90° rotated from the *left* panel around the vertical axis. The distance between the nitrogen of lanoconazole and of the heme iron, and the position of the 4′-hydroxylation site of diclofenac, are indicated in the *left* panel. The distances between the 4′-OH site of diclofenac and the heme iron and between the most proximal atom of diclofenac and the heme iron are indicated in the *right* panel. *C*, Arrangement of the chlorophenyl groups of diclofenac and lanoconazole. The distances between the chloride of the dichlorophenyl group of diclofenac and the center of the chlorophenyl ring of lanoconazole, and between the chloride of the chlorophenyl group of lanoconazole and the center of the dichlorophenyl ring of diclofenac, are indicated in the figure. *D*, interaction between diclofenac and Arg73. The distances between the carboxylate oxygens of diclofenac and the side-chain nitrogen of Arg73 are indicated in the figure.

The X-ray crystal structure of CYP105A1 R84A co-complexed with diclofenac and lanoconazole was determined at 2.1 Å resolution (Table S1). The m*F*_o_-D*F*_c_ polder omit map of diclofenac or lanoconazole indicates that diclofenac and lanoconazole both bind in the binding pocket (Fig. 5*A*). Lanoconazole was observed as a single conformation, and the nitrogen (position 3) of the imidazole group of lanoconazole was bound to the heme iron with a coordinate bond distance of 2.09 Å (Fig. 5*B*). In contrast, diclofenac is located distant from the heme group, with the 4′-hydroxylation site and the most proximal atom being ∼14 Å and ∼7.4 Å from the heme iron, respectively. The bound diclofenac forms a close contact with the co-bound lanoconazole. The chloride of the chlorophenyl group of lanoconazole is 3.33 Å from the center of the dichlorophenyl ring of diclofenac, and one chloride of the dichlorophenyl group of diclofenac is 3.87 Å from the center of the chlorophenyl ring of lanoconazole (Fig. 5*C*). This indicates the formation of Cl-π interactions between diclofenac and lanoconazole. The carboxylate oxygens of diclofenac are 3.01 Å and 2.97 Å from the side chain N^η1^ and N^η2^ atoms of Arg73 (Fig. 5*D*), showing formation of charged hydrogen bonds between diclofenac and Arg73.

**Figure 6:**
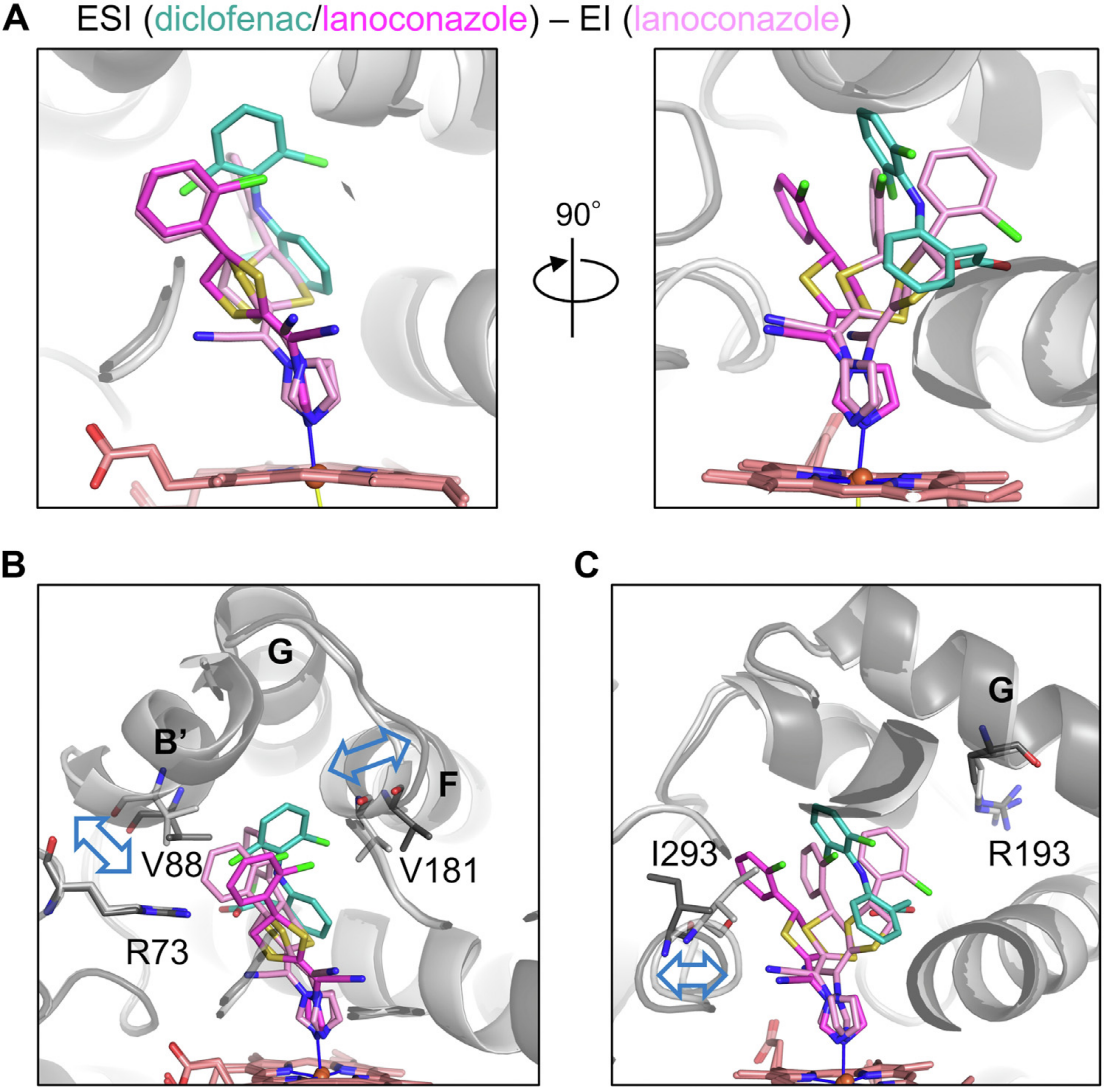
**Structural comparison between the ESI (diclofenac/lanoconazole) and EI (lanoconazole) complexes.***A*, close-up views of the substrate and inhibitor binding site. The *right* panel is the view 90° rotated from the *left* panel around the vertical axis. Diclofenac (*blue-green*) and lanoconazole (*magenta*) in the ESI complex and lanoconazole (*pink*) in the EI complex are shown as sticks. *B*, differences in the B′ and F helices. Positional differences between the ESI (*dark gray*) and EI (*light gray*) are indicated by *blue* double arrows. Arg73, Val88, and Val181 are shown as sticks. The distance of Val88 C^α^ atoms is 1.63 Å, and that of Val181 C^α^ atoms is 1.74 Å. *C*, differences in the loop (Ala289-Gly295). The positional difference between the ESI (*dark gray*) and EI (*light gray*) is indicated by a *blue* double arrow. Arg193 and Ile293 are shown as sticks. The distance of Ile293 C^α^ atoms is 1.72 Å

Comparison of the ESI and EI complexes shows several lanoconazole binding modes to the heme iron (Fig. 6*A*). The diclofenac binding site overlaps with the positions of the dithiolane and chlorophenyl groups of lanoconazole in the EI complex. This overlap of the binding site requires a positional change of the dithiolane and chlorophenyl groups between the ESI and EI complexes. The diclofenac binding site also overlaps with the methoxy-phenyl-piperazine part of ketoconazole (Fig. S9*A*) and with the dichlorophenyl groups of miconazole of the EI complexes (Fig. S9*B*).

Positional differences in the B′ and F helices were observed between the ESI and EI (lanoconazole) complexes (Fig. 6*B*). In contrast to these two helices, the position of Arg73 is similar in the ESI and EI complexes. The loop containing Ile293 is positioned similarly in the EI complexes (Fig. 4*D*), whereas the loop is positioned differently in the ESI and EI complexes (Fig. 6*C*) due to steric effects of the chlorophenyl group of lanoconazole.

### Fragment molecular orbital analysis of interactions in the ESI complex

We determined the specific interactions in the ESI complex by performing *ab initio* fragment molecular orbital (FMO) calculations (26). The evaluated inter-fragment interaction energies (IFIEs) (27) between specific CYP residues, heme, lanoconazole, and diclofenac in the ESI complex are shown in Figure S9. The IFIE between lanoconazole and the heme + Cys355 fragment was −377.3 kcal/mol, while the IFIE between lanoconazole and Arg73 was 12 kcal/mol (Fig. S10*A*). The other CYP residues formed no significant interactions with lanoconazole. The calculations confirmed that the heme group of CYP contributes greatly to lanoconazole binding. Additionally, Figure S10*A* highlights that lanoconazole interacts strongly (−27.4 kcal/mol) with diclofenac, indicating that diclofenac significantly stabilizes the ESI complex (Fig. S11*A*). The IFIEs between diclofenac and the CYP residues and between diclofenac and the heme group are shown in Figure S10*B*. Negatively charged diclofenac formed strong, attractive interactions with positively charged Arg73 and Arg193. Our FMO results also emphasize that diclofenac interacts strongly (−21.8 kcal/mol) with the crystal water molecule shown in the X-ray structure (Fig. S11*B*).

We determined the origin of these specific interactions in the ESI complex by conducting a pair interaction energy decomposition analysis (PIEDA) (28). The IFIEs evaluated using FMO calculations were decomposed into four energy components: electrostatic, exchange repulsion (EX), charge transfer with a mixed term (CT + mix), and dispersion (DI) contributions. As shown in Table 2, the interactions between negatively charged diclofenac and the positively charged arginine residues (Arg73 and Arg193) of CYP are mainly encompassed by the electrostatic term. The Arg73 side chain has strong electrostatic interactions with the carboxylate group of diclofenac, forming the salt bridge between diclofenac and Arg73 shown in Figure 5*D*. In contrast, the interaction between lanoconazole and diclofenac significantly contributes to DI, suggesting interactions with π-electrons, consistent with the crystal structure of the ESI complex (Fig. 5*C*) showing Cl-π interactions between lanoconazole and diclofenac.

**Table 2:** Total IFIEs (kcal/mol) for each pair of fragments were decomposed using PIEDA (28) into four energy components; electrostatic, EX, CT + mix, and DI contributions

	Total	Electrostatic	EX	CT + mix	DI
Lanoconazole and heme	−377.2	−584.3	788.6	−563.8	−17.7
Lanoconazole and diclofenac	−27.4	−21.7	16.5	−5.2	−17.0
Diclofenac and Arg73	−119.0	−117.3	9.8	−5.6	−5.9
Diclofenac and Arg193	−39.9	−39.9	0.0	0.0	0.0
Diclofenac and water	−21.8	−31.3	16.6	−5.6	−1.5

## Discussion

Previous mutation studies showed that the residues in the B′, F, G, and I helices of CYP105A1 affect the hydroxylation activities of the enzyme (Fig. S6*B*) (18, 19, 29). These residues are also located close to bound inhibitors (Fig. S6*A*). Our results indicate that these residues are important for recognizing diverse compounds and for regulating the catalytic reaction. Similar residues are involved in interactions with the inhibitors (Fig. S6*A*), whereas the *K*_d_ value of ketoconazole is approximately 10 times larger than those of lanoconazole and miconazole (Table 1). The molecular weight of ketoconazole is larger than that of lanoconazole or miconazole (Fig. 1). The structures of the EI complexes show that ketoconazole binds to the heme iron in a single conformation, whereas lanoconazole and miconazole each bind in two conformations (Fig. S3). Thus, the allowed conformation of ketoconazole for binding to the heme in the binding pocket is highly restricted compared with those of lanoconazole and miconazole. In addition, the binding pocket must undergo structural changes to accommodate ketoconazole, including positional changes of the F helix (Fig. 4*B*) and in His246 and surrounding residues (Figs. 4*C* and S8*B*). The structural properties of ketoconazole show that the entropic cost of binding to the heme is larger for ketoconazole than for lanoconazole and miconazole (30, 31). Therefore, ketoconazole showed a lower affinity (larger *K*_d_ value) than the other two inhibitors.

Our enzyme inhibition analyses demonstrated that lanoconazole acts as a noncompetitive inhibitor of CYP105A1 (Fig. 2). This result was surprising, as we had assumed that lanoconazole would inhibit activity by coordinating the nitrogen atom of its imidazole group to the heme iron. In noncompetitive inhibition, the inhibitor is typically believed to bind far from the substrate binding site, so a binding mode where the inhibitor coordinates to the heme iron is considered unlikely. Noncompetitive inhibition is characterized by the formation of an ESI complex and so we investigated the ESI complex using X-ray crystallography. We observed both the substrate (diclofenac) and the inhibitor (lanoconazole) in the binding pocket, with lanoconazole bound to the heme iron (Fig. 5). This binding mode differs from the generally predicted model for noncompetitive inhibitors, in which the inhibitor binds to an allosteric site of the enzyme. The ESI complex described in this work could form because the size of the binding pocket is optimal for the simultaneous entrance of diclofenac and lanoconazole, and because lanoconazole can bind to the heme iron in several orientations (Fig. 6*A*). In contrast to lanoconazole, ketoconazole and miconazole are competitive inhibitors of CYP105A1 (Fig. 2). Ketoconazole and miconazole form hydrophilic interactions with amino acid residues in the binding pocket (Fig. 3, *A* and *C*). Superimposing the imidazole of ketoconazole or miconazole onto that of lanoconazole in the ESI complex forces other parts of ketoconazole or miconazole to collide with the binding pocket amino acid residues and with diclofenac in the ESI complex (Fig. S12). These structural features prevent the formation of an ESI complex, and thus ketoconazole and miconazole act as competitive inhibitors of CYP105A1.

**Figure 7:**
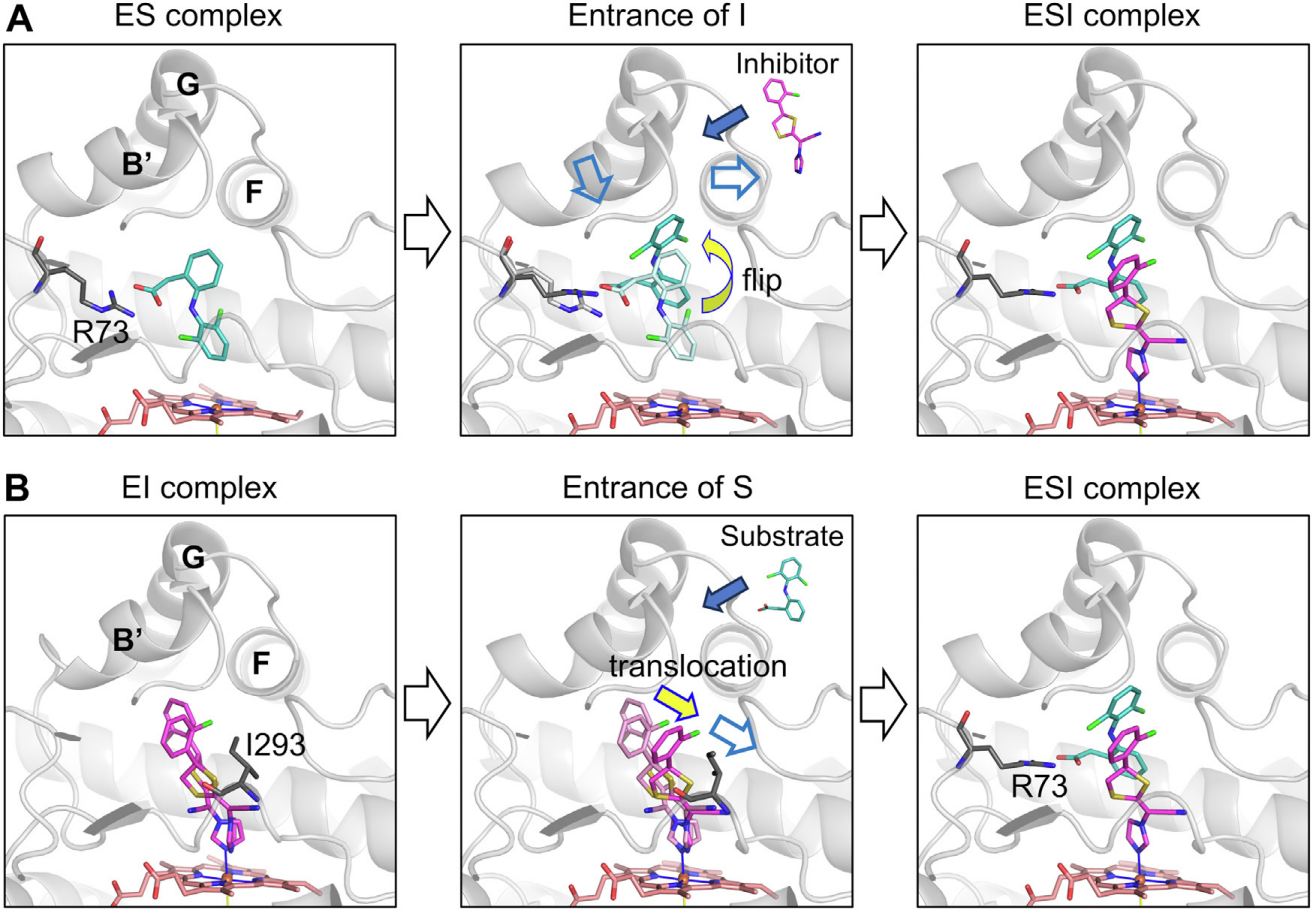
**Models explaining cooperative inhibition between a substrate and an apparent noncompetitive inhibitor.***A*, model for the formation of an ESI complex from an ES complex. The entrance of an inhibitor (lanoconazole) into the binding pocket of the ES complex affects the positions of the B′ and F helices (*blue* arrows in the center panel). These positional changes induce the substrate to flip (*yellow* arrow), and lanoconazole can bind to the heme iron to form the ESI complex. *B*, model for the formation of an ESI complex from an EI complex. The entrance of a substrate (diclofenac) into the binding pocket of the EI complex induces the inhibitor and the loop containing Ile293 to translocate (*yellow* and *blue* arrows in the center panel). These positional changes enable diclofenac to enter the pocket, and its position is stabilized by interactions with lanoconazole and Arg73 to form the ESI complex.

Our FMO calculations show that Cl-π and electrostatic interactions between diclofenac and lanoconazole help stabilize the ESI complex (Fig. S11*A*, Table 2). Furthermore, the position of the carboxylate group of diclofenac is stabilized by electrostatic interactions with Arg73, Arg193, and a water molecule (Fig. S11*B*, Table 2). A structure of the dichlofenac/CYP105A1 ES complex showed that diclofenac forms a salt bridge with Arg73, and that the 4′-hydroxylation site is located near the heme iron (Fig. S13*A*) (32). The distances and atomic properties of these interactions indicate that the interaction between diclofenac and heme in the ES complex is weaker than that between lanoconazole and heme (Fig. S13*B*). In addition, the entrance of lanoconazole into the binding pocket of the ES complex can cause diclofenac to flip around its carboxylate group because entry requires positional changes of the B′ helix and the F-G loop. This helix and loop are believed to form a narrow tunnel connecting the binding pocket to the protein surface. The described positional changes provide space for diclofenac in the ESI complex (Fig. S13*C*). The flipping of diclofenac allows lanoconazole to bind to the heme iron following the change in the position of the loop containing Ile293 (Fig. S13*D*). The flip around Arg73 and the conformational change in diclofenac (Fig. S13*E*) allow formation of the stable ESI complex (Fig. 7*A*).

As shown in Figure S14 of the supporting information, noncompetitive inhibition requires three conditions (1): the noncompetitive inhibitor has the same affinity for the enzyme (E) and for the enzyme-substrate (ES) complex, *K*_i_ = *K*’_i,_ (2) the substrate has the same affinity for the enzyme (E) and for the enzyme-inhibitor (EI) complex, *K*_s_ = *K*’_s_, and (3) the formed ESI complex cannot catalyze a reaction to form product. This inhibition mechanism is consistent with lanoconazole having the same affinity for E and the ES complex. The binding of lanoconazole does not compete with the binding of diclofenac in the ES complex because the flipping of diclofenac opens the binding site for lanoconazole, and the Cl-π and electrostatic interactions formed with diclofenac stabilize the binding of lanoconazole. Therefore, the apparent affinities of lanoconazole are the same for E and ES. The EI and ESI crystallographic structures show that the binding pocket allows lanoconazole to bind to the heme group in various orientations (Fig. 6*A*). This orientational flexibility also suggests that the entrance of diclofenac into the binding pocket of the EI complex causes the translocation of lanoconazole, and diclofenac is trapped by Arg73 to form the ESI complex (Fig. 7*B*). These structural properties ensure that diclofenac has the same affinity for E and EI. The structure of the ESI complex clearly demonstrated that the substrate is located far from the heme iron (Fig. 5), making hydroxylation at the 4′ position impossible, consistent with the third condition for noncompetitive inhibition.

The totality of our biochemical, structural, and computational analyses indicates that lanoconazole is not a typically defined noncompetitive inhibitor but that it appears to act as a noncompetitive inhibitor in the inhibition of diclofenac hydroxylation. The ESI complex with this apparent noncompetitive inhibitor is the first experimental structure of an ESI complex comprising CYP105A1, diclofenac, and lanoconazole. Previous structural analyses showed that the substrate binding pockets of several CYP enzymes, including CYP3A4 (33, 34), CYP2C8 (35) and CYP24A1 (36), can accommodate several compounds simultaneously. The structural study of CYP3A5 demonstrated that two molecules of azamulin, a CYP3A inhibitor, show the homotropic cooperative binding in the active site (37). Cholesterol, a substrate of CYP3A4, reportedly functions as a noncompetitive inhibitor of some substrates in CYP3A4 (38). A model explaining inhibition by cholesterol proposes that cholesterol binds near the substrate binding pocket of CYP3A4. It is therefore highly probable that cooperative inhibition achieved by interactions between the substrate, inhibitor, and amino acid residues, as observed in CYP105A1, also occurs in other CYP family proteins. Our findings on cooperative inhibition will widen the drug discovery search space for CYP enzyme inhibitors.

## Experimental procedures

### Materials

Ketoconazole, lanoconazole, and diclofenac were purchased from FUJIFILM Wako Chemicals, Ltd, Pharmaceutical and Medical Device Regulatory Science Society of Japan, and FUJIFILM Wako Chemicals, Ltd, respectively. Miconazole was purchased from LKT Laboratories, Inc. or the USP Convention. Nicotinamide adenine dinucleotide phosphate (NADPH) was purchased from Nacalai Tesque, Inc. NADPH-ferredoxin reductase and ferredoxin from spinach were purchased from Sigma-Aldrich. Glucose dehydrogenase and catalase were purchased from Fujifilm Wako Chemicals, Ltd. All other chemicals used were commercially available and of the highest quality.

### Enzyme inhibition assay

Expression and purification of the CYP105A1 variant R84A were performed as previously described (18, 19). Purified enzyme was used to calculate *K*_d_, while crude enzyme before column purification was used to measure inhibitory activity. *K*_d_ values between CYP105A1 R84A and each inhibitor were determined as follows. 100 mM potassium phosphate (KPi) (pH 7.5) buffer solutions containing 0.2 μM purified CYP105A1 R84A and each inhibitor (2, 5, 10, and 20 μM for ketoconazole; 1, 2, 5, and 10 μM for lanoconazole; 1, 2, 5, and 10 μM for miconazole) were prepared. Each inhibitor was dissolved in ethanol and added at a final ethanol concentration of 0.5%. For each solution, enzyme-inhibitor binding difference spectra were measured in the 350 to 500 nm wavelength range using a Hitachi U-3310 spectrophotometer with a head-on photomultiplier (Tokyo, Japan) as previously described (39). A buffer solution containing 0.2 μM P450 and 0.5% ethanol was used as a control. The difference in absorbance between *λ*_max_ and *λ*_min_ in the resulting enzyme-inhibitor binding difference UV spectrum was plotted on the y-axis and inhibitor concentration on the x-axis, and *K*_d_ was calculated using Kaleida-Graph (Synergy software, Perkiomen Ave). Three separate experiments were performed to obtain *K*_d_ values.

Inhibitory activity of each inhibitor on hydroxylation activity of CYP105A1 variant R84A toward diclofenac was measured in the following reconstituted system: 50 μl of reaction solution consisted of 1% glucose, 1 U/ml glucose dehydrogenase, 0.1 mg/ml catalase, 0.1 mg/ml spinach ferredoxin, 0.1 U/ml spinach NADPH-ferredoxin reductase, 1 mM NADPH, 20 nM P450, 2.5, 5, 10, 30 μM diclofenac, and inhibitors (0, 5, 10, 20 μM for ketoconazole; 0, 0.5, 1, 2 μM for lanoconazole; 0, 0.5, 1, 2, 5 μM for miconazole) in 120 mM Tris-HCl (pH 7.4) buffer containing 1 mM EDTA. Ethanol concentration in the final reaction solution was 0.8%. Reaction was carried out at 30°C for 0 min and 15 min for ketoconazole, 0 min and 8 min for lanoconazole, and 0 min and 10 min for miconazole. After each interval, the reaction was stopped by an addition of 50 μl of acetonitrile into each reaction solution. The solution was centrifuged at 10,000 x *g* for 15 min, and the resultant supernatant was analyzed by ultra performance liquid chromatography (UPLC) under the following conditions: apparatus, H-Class (Waters Co.); column, ACQUITY UPLC BEH C18 (1.7 μm, 2.1 × 100 mm) (Waters Co.); UV detection, 276 nm; column temperature, 40^o^C; flow rate, 0.7 ml/min; injection volume, 10 μl; mobile phase, a linear gradient of 25 to 43% acetonitrile in aqueous solution containing 0.1% trifluoroacetic acid (TFA) for 3 min, followed by 43 to 58% acetonitrile in aqueous solution containing 0.1% TFA for 1 min, followed by 58 to 100% acetonitrile in aqueous solution containing 0.1% TFA for 2 min. The *k*_obs_, *i*.*e*., *v* (μM/min)/[E] was calculated from the recovery rate of 4′-hydroxydiclofenac, which is a metabolite of diclofenac, and the concentration (μM) of CYP105A1 R84A. Two separate experiments were performed to obtain the *k*_obs_ value for 30 μM diclofenac in 5 μM miconazole. Three separate experiments were performed to obtain all other *k*_obs_ values. Lineweaver-Burk plot and Dixon plot were prepared from the obtained *k*_obs_ for each reaction. Kinetic parameters (*K*_i_) of ketoconazole, lanoconazole, and miconazole were calculated by Dixon-plot.

### Crystallization, diffraction data collection, and structure refinement

Overexpression and purification procedures for crystal preparation were followed as previously reported (18, 19), but the purification procedure was slightly modified as follows. Purification of CYP105A1 R84A was performed by Ni-affinity, anion exchange, and size-exclusion chromatography steps. Inhibitors (ketoconazole, lanoconazole, and miconazole) were dissolved in ethanol at a concentration of 10 mM. For EI complex preparation, each inhibitor was added to the purified enzyme in a 10-fold molar excess with a final ethanol concentration of less than 1%. The EI complexes were incubated at 4°C for 1 day. Before crystallization experiments, an excess amount of inhibitor was removed, and the EI complex solution was concentrated to approximately 0.4 mM by using AmiconUltra 10-kDa molecular-weight cutoff filters (Merck, USA). Crystallization experiments were performed by the hanging-drop vapor diffusion method at 10°C. Crystals of EI complexes were obtained using a reservoir solution consisting of 20% to 28% (*w*/*v*) polyethylene glycol (PEG) 2000 monomethyl ether (MME), 50 mM Bis-Tris pH 6.0 or 6.5, 0.2 M NaCl. Substrate (diclofenac) was dissolved in ethanol at a concentration of 10 mM. For ESI complex preparation, enzyme, diclofenac, and lanoconazole were mixed in a molar ratio of 1:5:5 (9.6 μM:48 μM:48 μM) with a final ethanol concentration of less than 1%. The ESI complex was incubated at 4°C for 1 day. Before crystallization experiments, an excess amount of substrate and inhibitor was removed, and ESI complex solution was concentrated to approximately 0.4 mM by using AmiconUltra 10-kDa molecular-weight cutoff filters. Crystallization experiments were performed by the hanging-drop vapor diffusion method at 10°C. Crystals of the ESI complex were obtained using a reservoir solution consisting of 26% (*w*/*v*) PEG2000MME, 50 mM Bis-Tris pH 6.5, 0.2 M NaCl.

Diffraction data sets for EI and ESI complexes were collected at the BL17A beamline of the Photon Factory (KEK). Crystals were flash frozen in liquid nitrogen, and the data collection was performed at 95 K using cold N_2_ gas equipped with the beamline. Diffraction intensities were integrated using the program *XDS* (40) and scaled using the program *Aimless* (41).

The initial phase for structure determination was obtained by the molecular replacement method using the program *Phaser* (42). The crystal structure of the substrate-free CYP105A1 R84A mutant (PDB ID: 2ZBY) (18) was used as a search model. Structure refinement was performed using the program *Phenix* (43). Structural refinement procedures of EI and ESI complexes were performed as follows. Several cycles of refinement were performed for atomic coordinates and atomic displacement parameters of the protein moiety, heme group, and water molecules. Geometric restraints for inhibitors and substrate were prepared using the program *PRODRG* (44). Then, inhibitors and substrate were fitted into electron density maps, and several cycles of refinement were performed for atomic coordinates and atomic displacement parameters of all atoms in the coordinate files. For the EI complexes, the (2*S*,4*R*)-enantiomer of ketoconazole was refined as a single conformation with an occupancy of 1.0, the (*R*)-enantiomer of lanoconazole was refined as two conformations with occupancies of 0.5 and 0.5, and the (*S*)-enantiomer of miconazole was refined as two conformations with occupancies of 0.5 and 0.5. For the ESI complex, a diclofenac and the (*R*)-enantiomer of lanoconazole were refined with occupancies of 1.0 and 1.0. Models and electron density maps were displayed and modified using the program *Coot* (45). Figures of structures and electron density maps were prepared using the program *PyMOL* (The PyMOL molecular graphics system, version 2.0, Schrödinger, LCC.).

### Fragment molecular orbital calculation

Electronic properties of the specific interactions between CYP residues, heme, lanoconazole, and diclofenac in the ESI complex were investigated using the *ab initio* FMO calculation (26). In FMO calculation, the target protein is divided into fragments, and the electronic properties of the target complex are estimated from the electronic properties of monomers and dimers of the fragments. We explicitly included 204 crystal water molecules in the X-ray crystal structure to properly consider the effect of water molecules on the specific interactions. The *ab initio* second-order Møller-Plesset (MP2) (46, 47) method of the FMO program *ABINIT-MP Open* Ver. One Rev. 22 (48) was used to accurately investigate π-π stacking, NH-π, and CH-π interactions as well as hydrogen-bonding and electrostatic interactions between each of the CYP residues, heme, lanoconazole, and diclofenac. The 6-31G basis set was used for the FMO calculations. From inter-fragment interaction energies (27) obtained by the FMO calculations, important CYP residues for binding between CYP, heme, lanoconazole, and diclofenac were highlighted at an electronic level. To elucidate the origin of the specific interactions in the ESI complex, we moreover conducted PIEDA (28). The IFIEs evaluated using FMO calculations were decomposed into four energy components: electrostatic, EX, CT + mix, and DI contributions.

As shown in the crystal structure of the ESI complex, heme iron is coordinated by the four nitrogen atoms of the pyrrole ring, the Cys355 thiolate of the enzyme, and the nitrogen of the imidazole group of lanoconazole. To precisely describe the coordinated state of the ESI complex, we considered heme and Cys355 as the same fragment in the present FMO calculations, in which +2 charge was assigned to heme iron, and deprotonated states for the two propionic acids of heme were employed.

## Data availability

The coordinates and structure factors have been deposited in PDB under the accession codes of 9KW2 (EI, ketoconazole), 9KW3 (EI, lanoconazole), 9KW4 (EI, miconazole), and 9KW5 (ESI).

## Supporting information

This article contains supporting information.

## Conflict of interest

The authors declare that they have no conflicts of interest with the contents of this article.
