# Supplementary material for: Cooperative inhibition in cytochrome P450 between a substrate and an apparent noncompetitive inhibitor
Source: J Biol Chem. 2025 Apr 15;301(6):108513. doi: 10.1016/j.jbc.2025.108513 (PMC12144444; doi:10.1016/j.jbc.2025.108513)
Supplement: Supporting Figures [file mmc1.pdf]

Supporting Information

Cooperative inhibition in Cytochrome P450 between a substrate and an apparent non-competitive inhibitor

Yu Hirano[a,b,#], Sachiyo Yoneda[c,#], Kaori Yasuda[c], Noriyuki Kurita[d], Fumihiro Kawagoe[e], Bunzo Mikami[f,g], Teisuke Takita[h], Kiyoshi Yasukawa[h], Shinichi Ikushiro[c], Midori Takimoto-Kamimura[i], Atsushi Kittaka[e], Toshiyuki Sakaki[c,], Taro Tamada[a,b,*]*

[a]Institute for Quantum Life Science, National Institutes for Quantum Science and Technology, Inage, Chiba 263-8555, Japan. [b]Quantum Life Science Course, Graduate School of Science and Engineering, Inage, Chiba 263-8522, Japan. [c]Graduate School of Engineering, Biotechnology and Pharmaceutical Engineering, Toyama Prefectural University, Imizu, Toyama 939-0398, Japan. [d]Department of Computer Science and Engineering, Toyohashi University of Technology, Toyohashi, Aichi 441-8580, Japan. [e]Faculty of Pharmaceutical Sciences, Teikyo University, Itabashi, Tokyo 173-8605, Japan. [f]Research Institute for Sustainable Humanosphere, Kyoto University, Uji, Kyoto 611-0011, Japan. [g]Institute of Advanced Energy, Kyoto University, Uji, Kyoto 611-0011, Japan. [h]Division of Food Science and Biotechnology, Graduate School of Agriculture, Kyoto University, Sakyo-ku, Kyoto 606-8502, Japan. [i]Quantum-Structural Life Science Laboratories, CBI Research Institute, Kyowa Create Daiichi build. 3F, 3-11-1 Shibaura Minato, Tokyo, 108-2234, Japan.

# Equal contribution

* Corresponding authors

Taro Tamada: tamada.taro@qst.go.jp

Toshiyuki sakaki: tsakaki@pu-toyama.ac.jp

Supporting Figures

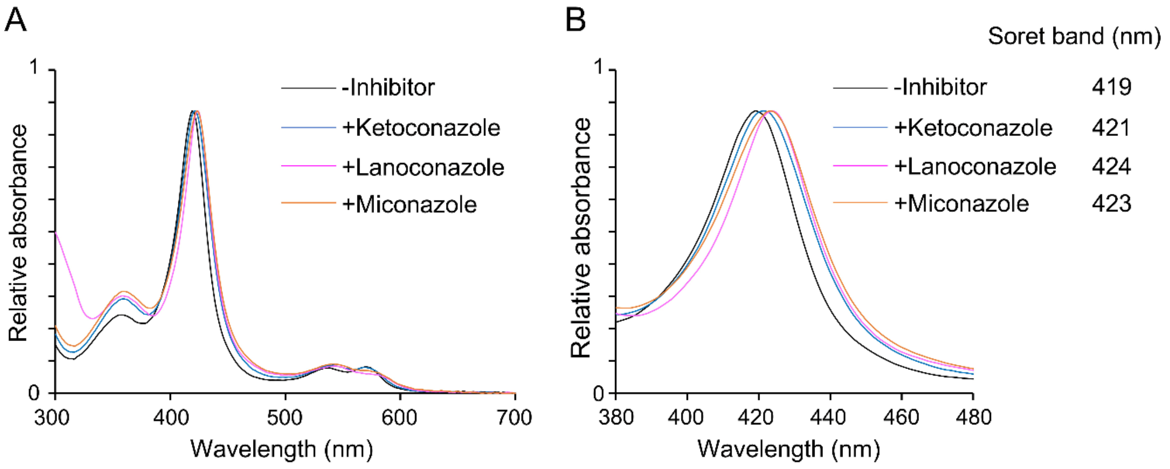

Figure S1. UV-visible absorption spectra. (A) Spectra for ferric form (black), ketoconazole complex (blue), lanoconazole complex (pink), and miconazole complex (orange) were plotted at the wavelength range from 300 nm to 700 nm. (B) Close-up view of (A) in the wavelength range from 380 nm to 480 nm. Wavelengths of Soret bands are indicated.

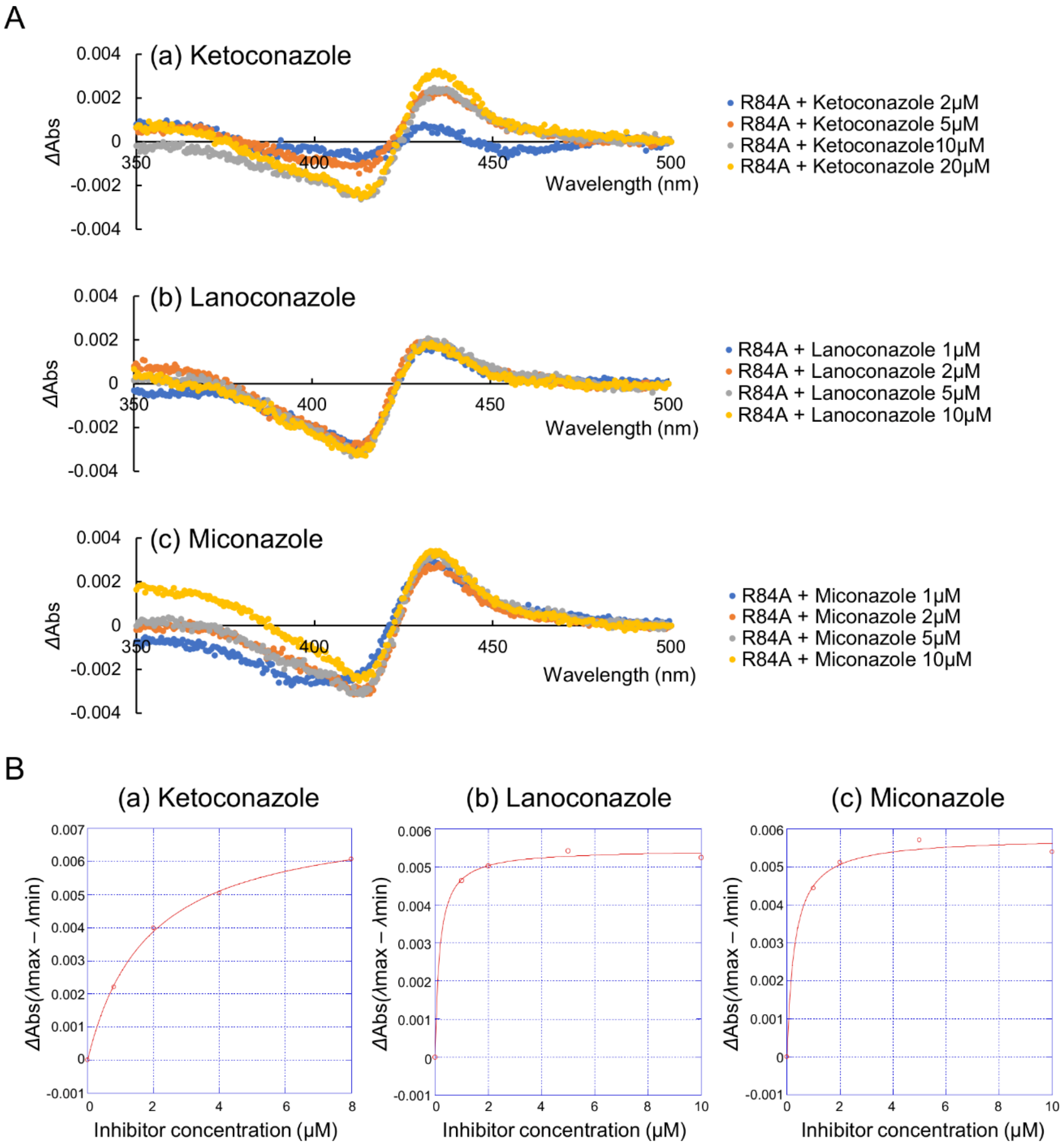

Figure S2. Enzyme inhibition assay. (A) Representative enzyme - inhibitor binding difference UV spectra in the presence of 0.2 μM CYP105A1 R84A and each inhibitor, (a) ketoconazole 2, 5, 10, 20 μM; (b) lanoconazole 1, 2, 5, 10 μM; (c) miconazole 1, 2, 5, 10 μM. (B) Plots of the difference in absorbance between λmax and λmin in the resulting enzyme - inhibitor binding difference UV spectrum.

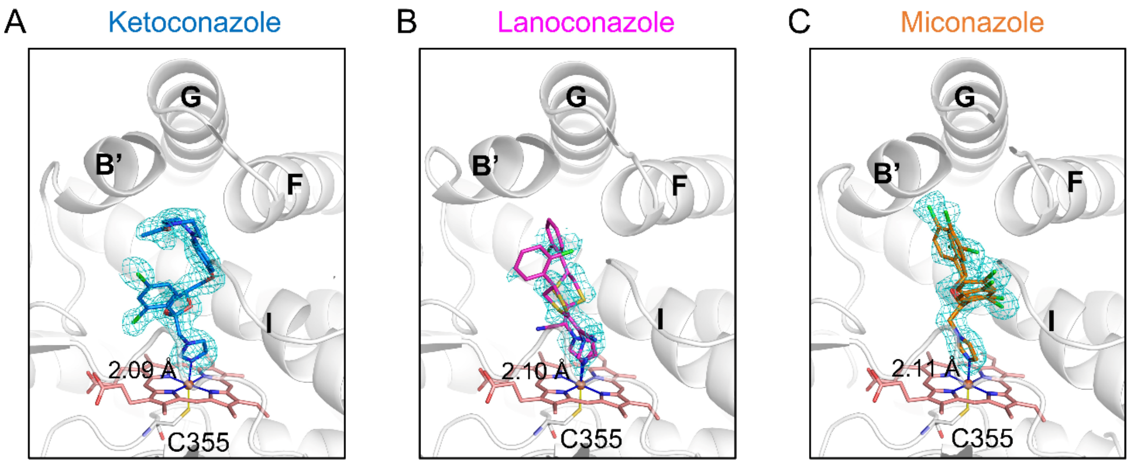

Figure S3. mF_o-DF_c polder omit map of the inhibitor in the EI complex. (A) The ketoconazole binding site, (B) the lanoconazole binding site, and (C) the miconazole binding site. mF_o-DF_c polder omit maps contoured at 3.0 σ levels are shown as light blue meshes. The inhibitors, heme groups, and Cys355 are shown as sticks. The positions of B', F, G, and I helices are indicated in the figures. Fe coordinate bonds are shown as thin lines. The distance between Fe and the nitrogen of the imidazole ring of each inhibitor is indicated.

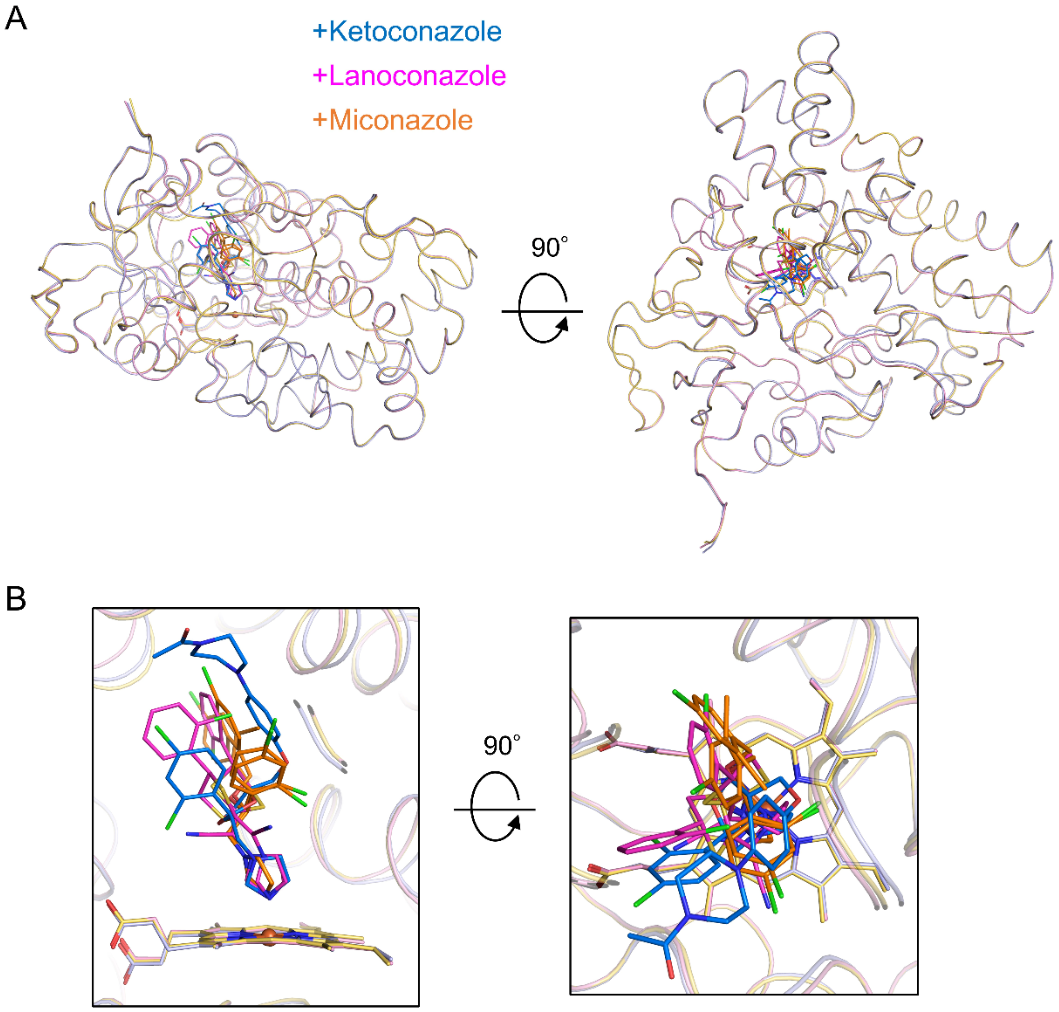

Figure S4. Superposition of the EI complex structures. (A) Overall structures, and (B) close-up view around the inhibitor binding sites. The ketoconazole, lanoconazole, and miconazole complexes are colored blue, pink, and orange, respectively. The inhibitors and heme groups are shown as sticks. The protein moieties are shown as ribbons. The left and right panels relate to 90° rotation from the horizontal axis.

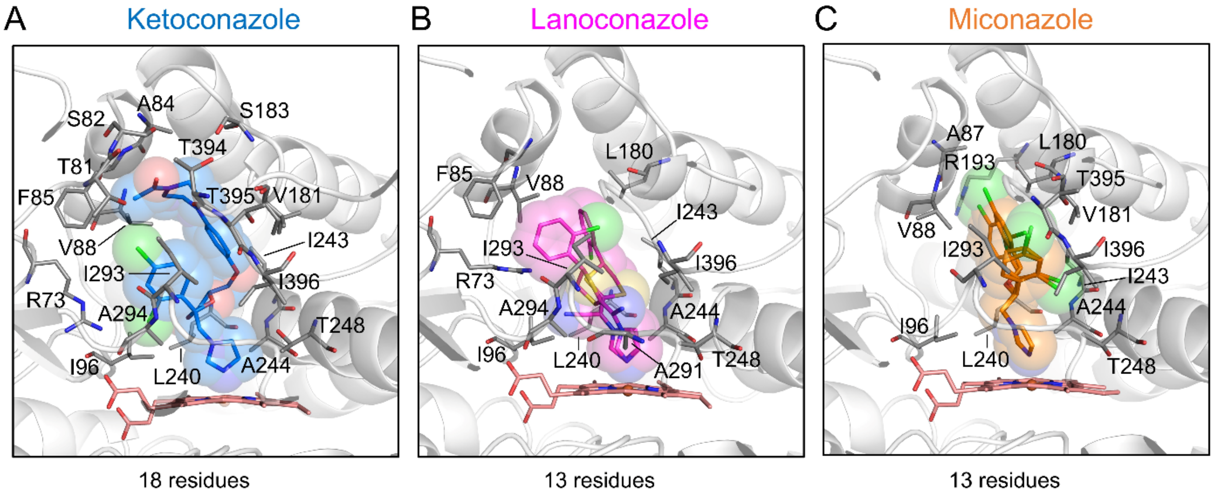

Figure S5. Binding pocket of the EI complexes. (A) The ketoconazole binding site, (B) the lanoconazole binding site, and (C) the miconazole binding site. Inhibitors are shown as sticks and van der Waals radii of the inhibitor atoms are transparently overlayed. Residues within 4 Å of each inhibitor are shown as gray sticks. The numbers of the residues within 4 Å of each inhibitor are indicated below the figures.

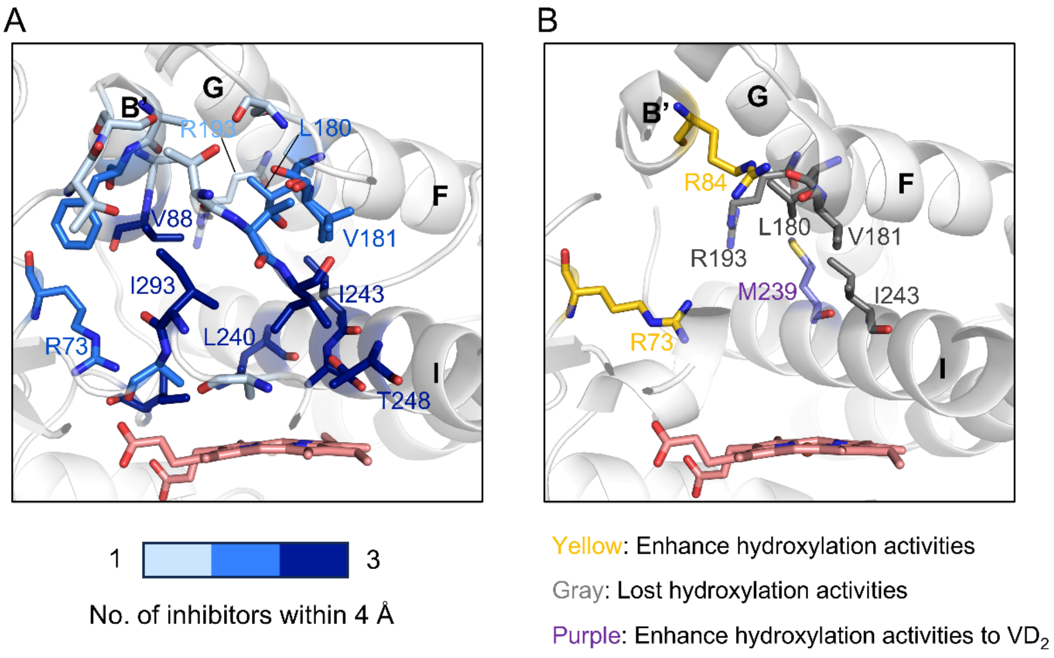

Figure S6. Residues forming the binding pocket of the EI complex. (A) Positions of the residues involved in the recognition of the inhibitors. The residues are colored according to the number of inhibitors within 4 Å from light blue (one inhibitor) to dark blue (three inhibitors). (B) Positions of the residues affected the hydroxylation activities of CYP105A1 (18, 19, 29). The residues are shown as sticks in the wild-type structure (PDB ID: 2ZBX) (19). Mutations of Arg73 and Arg84 (yellow) enhanced the hydroxylation activities. Mutations of Leu180, Val181, Arg193, and Ile243 (gray) lost the hydroxylation activities. The mutation of Met239 (purple) enhanced the hydroxylation activity toward VD_2.

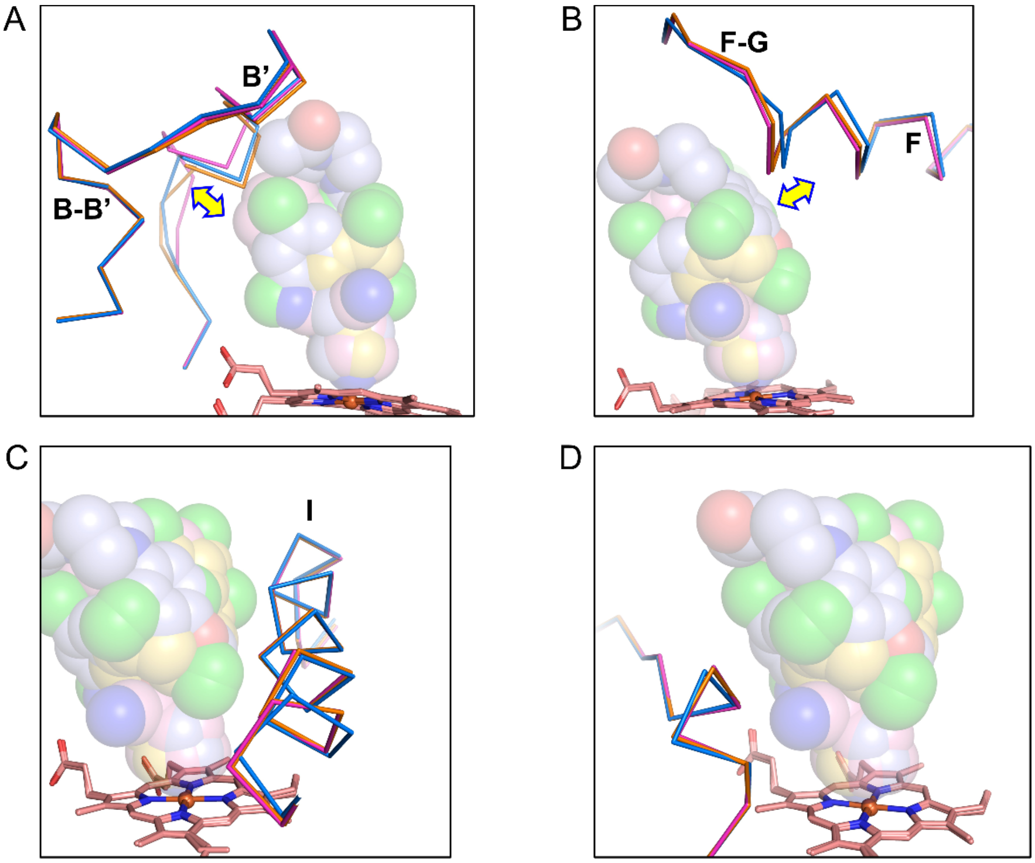

Figure S7. Structural comparison of the binding pocket amongst the EI complexes. (A) The B' helix and B-B' loop, (B) the F helix and F-G loop, (C) the I helix, and (D) the loop (Ala289-Gly295). The ketoconazole, lanoconazole, and miconazole complexes are colored blue, pink, and orange, respectively. Inhibitors are shown as CPK models. Structural differences in the B' and F helices are indicated by yellow arrows in (A) and (B).

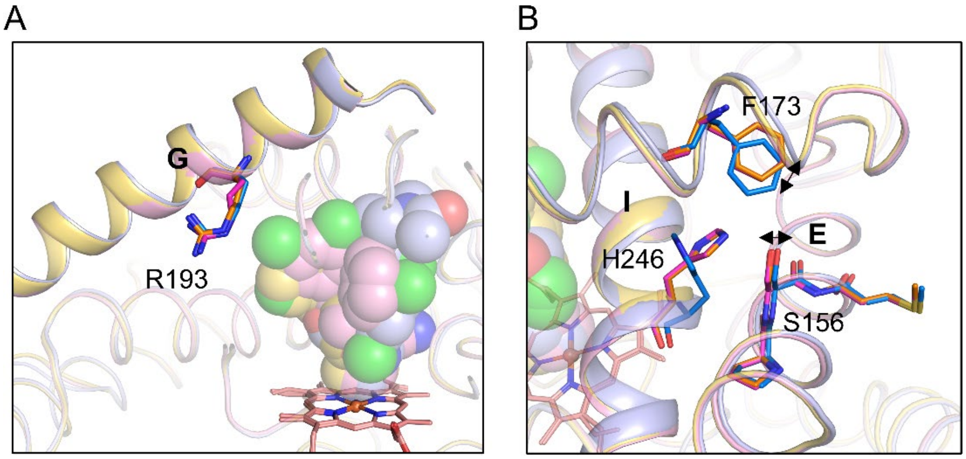

Figure S8. Structural comparison amongst the EI complexes. (A) The G helix and (B) the residues near His246. The ketoconazole, lanoconazole, and miconazole complexes are colored blue, pink, and orange, respectively. Inhibitors are shown as CPK models and amino acid residues indicated in the figures are shown as sticks. Structural differences in the Phe173 and the E helix are indicated by black double arrows in (B). The distances of Phe173 C^{ζ} atom are 1.31 Å (ketoconazole-lanoconazole) and 1.31 Å (ketoconazole-miconazole), and those of Ser156 C^{α} atom are 0.38 Å (ketoconazole-lanoconazole) and 0.36 Å (ketoconazole-miconazole).

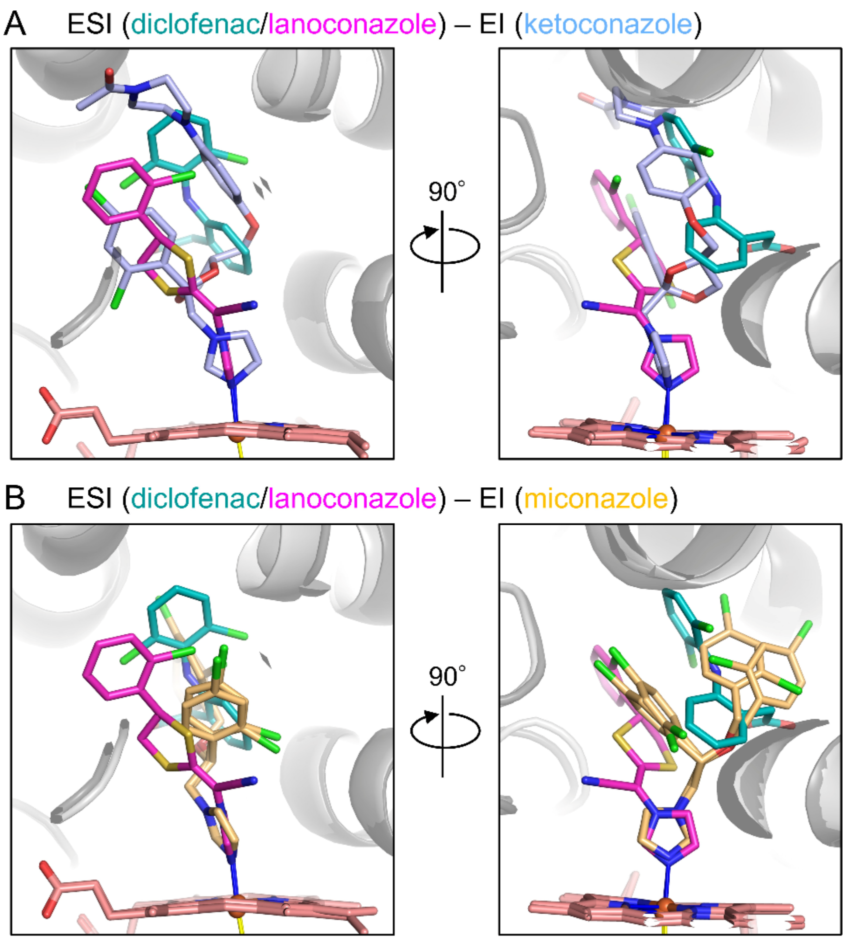

Figure S9. Structural comparison between the ESI and EI complexes. (A) The ESI (diclofenac/lanoconazole) and EI (ketoconazole) binding sites and (B) the ESI (diclofenac/lanoconazole) and EI (miconazole) binding sites. The right panel is the view 90° rotated from the left panel around the vertical axis. Diclofenac (blue-green) and lanoconazole (magenta) in the ESI complex and ketoconazole (light blue) and miconazole (light orange) in the EI complexes are shown as sticks.

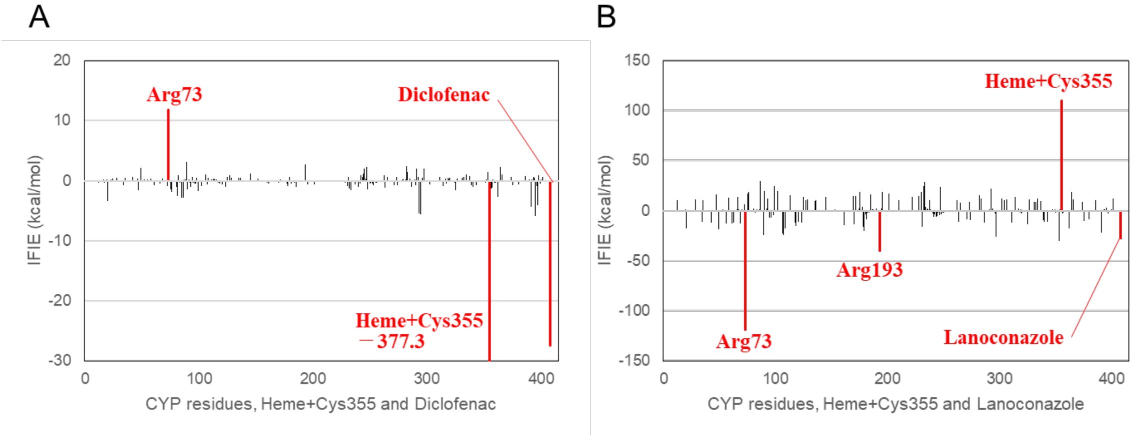

Figure S10. IFIEs in the ESI complex. (A) IFIEs of lanoconazole with CYP residues, heme+Cys355, and diclofenac, and (B) IFIEs of diclofenac with CYP residues, heme+Cys355, and lanoconazole.

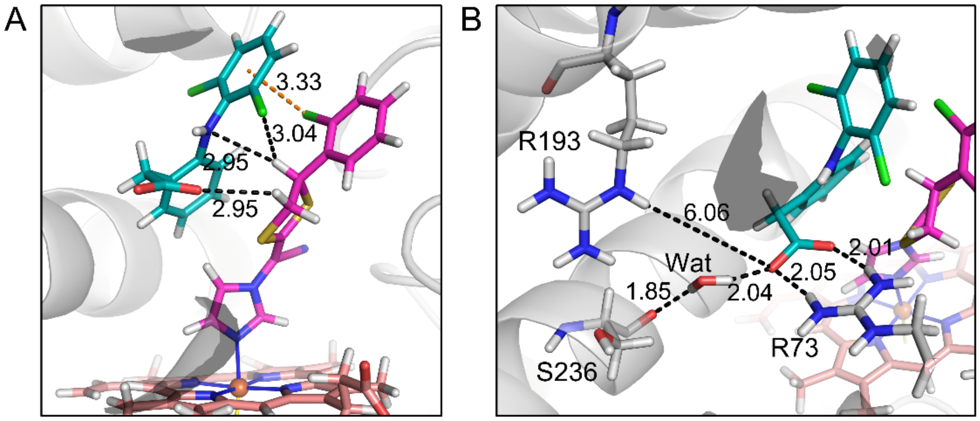

Figure S11. The FMO analysis of interactions in the ESI complex. (A) Interactions between diclofenac and lanoconazole. Cl-π and electrostatic interactions are shown as orange and black dotted lines, respectively, with the distances in Å. (B) Electrostatic interactions involved in the carboxylate group of diclofenac. Arg73, Arg193, and a water molecule that forms a hydrogen bond with the main-chain carbonyl oxygen of Ser236 make electrostatic interactions with the carboxylate group of diclofenac. The distances involved in the interactions are shown in Å.

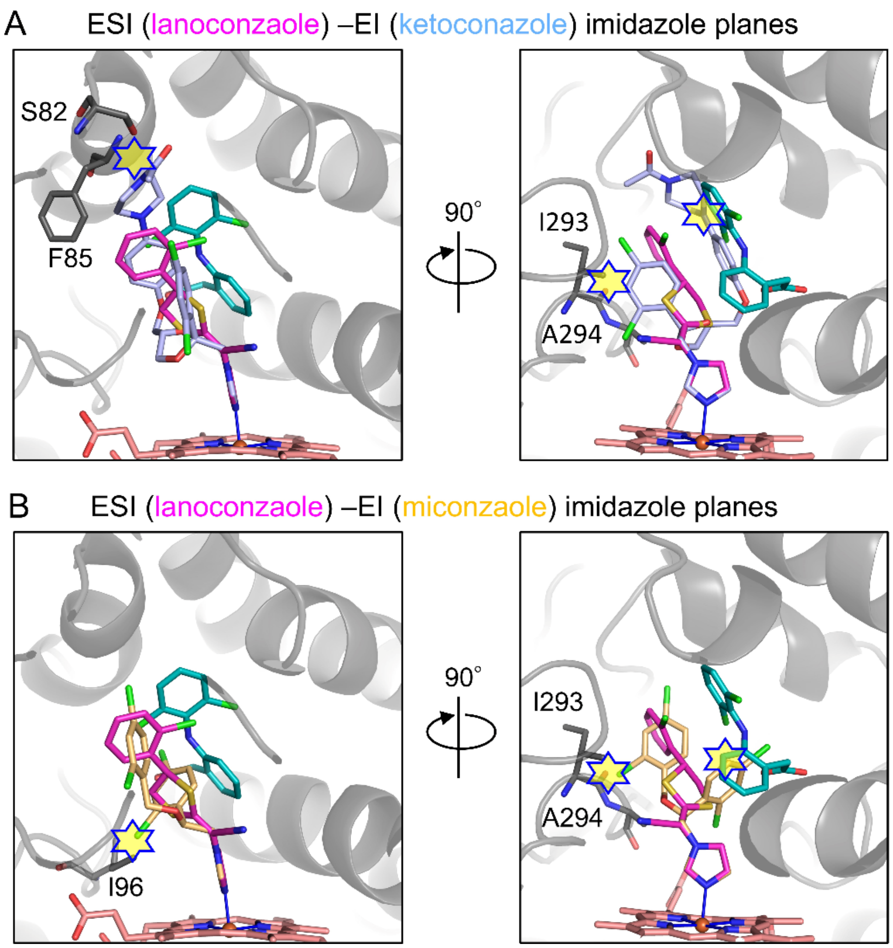

Figure S12. Superposition of the imidazole planes of the inhibitors. (A) Superposition of the imidazole plane of ketoconazole in the EI complex with that of lanoconazole in the ESI complex. (B) Superposition of the imidazole plane of miconazole in the EI complex with that of lanoconazole in the ESI complex. A conformation of ketoconazole or miconazole is shown in each figure. The ketoconazole and miconazole make collisions with amino acid residues and diclofenac as indicated by stars in the figures. When the ketoconazole or miconazole is rotated around the imidazole plane, the inhibitors also make collisions with amino acid residues and diclofenac.

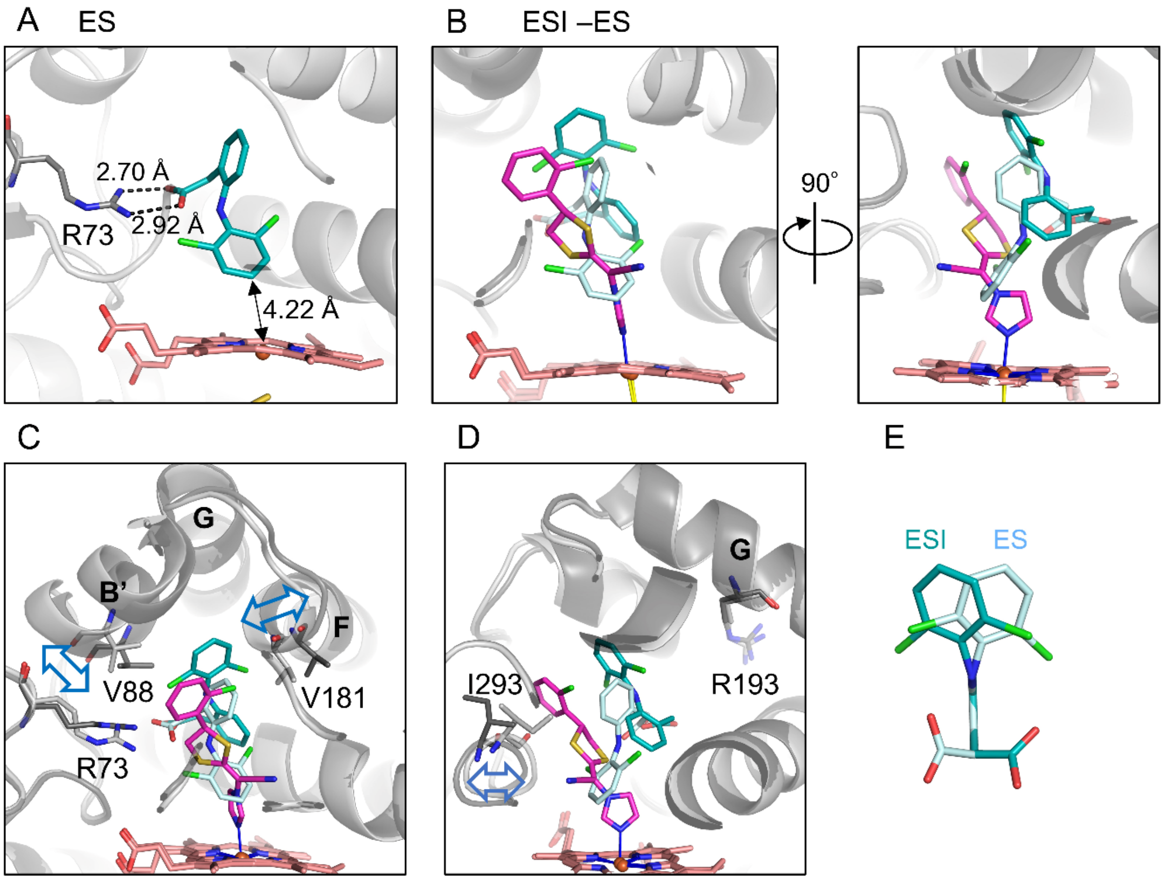

Figure S13. Structural comparison between the ESI and ES complexes. (A) The substrate binding site in the ES complex (PDB ID: 9JI6) (32). Distances between the side chain of Arg73 and the carboxylate group of diclofenac and that between the 4'-hydroxylation site of diclofenac and the heme iron are indicated. (B) Superposition of the ESI and ES complexes. Diclofenac (blue-green) and lanoconazole (magenta) in the ESI complex and diclofenac (cyan) in the ES complex are shown as sticks. (C) Differences in the B' and F helices. Positional differences between the ESI (dark gray) and ES (light gray) are indicated by blue double arrows. The distance of Val88 Cα atoms is 1.84 Å, and that of Val181 Cα atoms is 1.66 Å. Arg73, Val88, and Val181 are shown as sticks. (D) A difference in the loop (Ala289-Gly295). The positional difference between the ESI (dark gray) and ES (light gray) is indicated by a blue double arrow. Arg193 and Ile293 are shown as sticks. The distance of Ile293 C^{α} atoms is 1.53 Å. (E) Conformational difference in diclofenac. Phenyl groups of diclofenac molecules were superposed between the ESI (blue-green) and ES (cyan) complexes. Positions of carboxylate and dichlorophenyl groups relative to phenyl groups show a difference between the ESI and ES complexes.

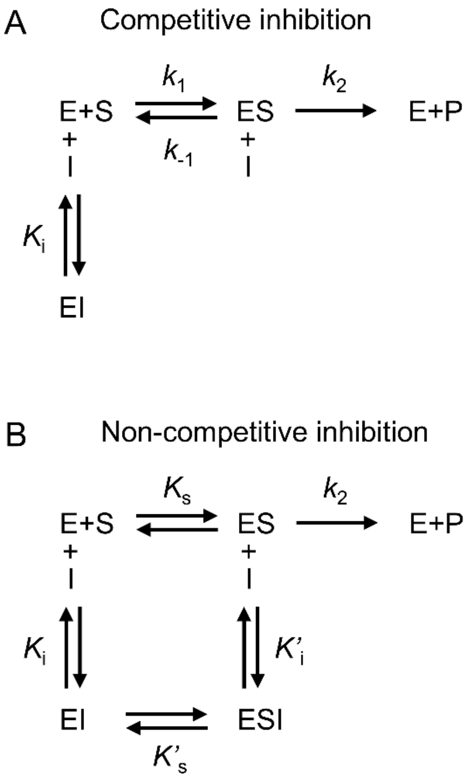

Figure S14. Scheme of enzyme inhibition. (A) Competitive inhibition, and (B) non-competitive inhibition, where K_s=K'_s, and K_i=K'_i.

Supporting Tables

Table S1. Diffraction data and refinement statistics.

	EI (ketoconazole)	EI (lanoconazole)	EI (miconazole)	ESI
Diffraction data				
Resolution (Å)	50-1.8 (1.84-1.80)	50-1.8 (1.84-1.80)	50-1.8 (1.84-1.80)	50-2.1 (2.16-2.10)
Space group	$P2_12_12_1$	$P2_12_12_1$	$P2_12_12_1$	$P2_12_12_1$
Unit cell (Å)	a = 52.73, b = 53.87, c = 142.01	a = 52.23, b = 53.49, c = 140.57	a = 52.29, b = 53.48, c = 140.05	a = 52.61, b = 53.56, c = 141.25
Observed reflections	258183 (14147)	250174 (14447)	251442 (13941)	159820 (11451)
Unique reflections	38402 (2208)	37416 (2167)	37286 (2156)	24098 (1925)
Completeness (%)	100.0 (99.7)	100.0 (99.7)	99.9 (99.2)	100.0 (99.9)
$I/\sigma(I)$	7.9 (2.6)	11.7 (2.7)	14.0 (3.3)	8.8 (1.8)
R_{meas}[a] (%)	13.5 (56.3)	11.2 (67.8)	8.4 (53.8)	12.6 (89.3)
$CC_{1/2}$	0.996 (0.897)	0.996 (0.869)	0.998 (0.914)	0.998 (0.815)
Refinement				
R_{work}[b]/R_{free}[c] (%)	19.5 (22.7)/ 23.5 (28.7)	18.1 (23.8)/ 22.6 (33.5)	18.4 (22.3)/ 22.8 (29.9)	21.2 (26.7)/ 25.5 (28.6)
R.m.s.d. bonds (Å)	0.006	0.006	0.007	0.003
R.m.s.d. angles (°)	0.896	0.883	0.850	0.568
Ramachandran (%) Favored/allowed/outlier	98.0/1.8/0.2	97.5/2.2/0.3	97.8/2.0/0.2	97.8/2.2/0.0

No. of atoms[d]				
Protein	3156	3188	3208	3143
Heme	43	43	43	43
Inhibitor	36	40	50	20
Substrate	-	-	-	19
Solvent	264	450	302	204
PDB code	9KW2	9KW3	9KW4	9KW5

Values in parentheses show the highest resolution shell.
[a]$R_{\text{meas}} = \Sigma_{hkl}(n/(n-1))^{1/2}\Sigma_i|I_{hkl,i}-\langle I_{hkl}\rangle|/\Sigma_{hkl}\Sigma i I_{hkl,i}$.
[b]$R_{\text{work}} = \Sigma_{hkl}||F_{\text{obs}}|-|F_{\text{calc}}||/\Sigma_{hkl}|F_{\text{obs}}|$
[c]R_{free} was calculated by using the 5% of the reflections that were not included in the refinement as a test set.
[d]Atoms in alternate conformation were counted separately.

Table S2. Root-mean-square-deviations (Å) in the superposition for 400 C^{α} atoms of the EI complexes

	Ketoconazole	Lanoconazole	Miconazole
Ketoconazole	-	-	-
Lanoconazole	0.35	-	-
Miconazole	0.30	0.31	-
